# Bioactive Metabolites From Acid-Tolerant Fungi in a Thai Mangrove Sediment

**DOI:** 10.3389/fmicb.2020.609952

**Published:** 2021-01-22

**Authors:** Hai Gao, Yanan Wang, Qiao Luo, Liyuan Yang, Xingxing He, Jun Wu, Konthorn Kachanuban, Pongthep Wilaipun, Weiming Zhu, Yi Wang

**Affiliations:** ^1^School of Medicine and Pharmacy, Ocean University of China, Laboratory for Marine Drugs and Bioproducts of Qingdao National Laboratory for Marine Science and Technology, Qingdao, China; ^2^School of Pharmaceutical Sciences, Southern Medical University, Guangzhou, China; ^3^Faculty of Fisheries, Kasetsart University, Bangkok, Thailand

**Keywords:** acid-tolerant fungi, Thai mangrove sediment, genetic relationship, secondary metabolites, bioactive diversity

## Abstract

Despite being potentially useful extremophile resources, there have been few reports on acid-tolerant fungi and their bioactive metabolites. Acidophilic/aciduric fungi (*n* = 237) were isolated from Thai mangrove sediments in an acidic medium. Using fungal identification technology (including morphologic observation, chemical screening, and sequence comparisons) all the isolates were identified and 41 representative isolates were selected for analysis of the phylogenetic relationships (ITS rDNA, β-tubulin, calmodulin, and actin gene sequences). There were seven genera identified – *Penicillium*; *Aspergillus*; *Talaromyces*; *Cladosporium*; *Allophoma*; *Alternaria*; and *Trichoderma* – in four taxonomic orders of the phylum Ascomycota, and *Penicillium*, *Aspergillus*, and *Talaromyces* were the dominant genera. Acidity tolerance was evaluated and 95% of the isolates could grow under extremely acidic conditions (pH 2). Six strains were classed as acidophilic fungi that cannot survive under pH 7, all of which had an extraordinarily close genetic relationship and belonged to the genus *Talaromyces*. This is the first report on the acidophilic characteristics of this genus. The antimicrobial, anti-tumor, and antiviral activities of the fermentation extracts were evaluated. Nearly three-quarters of the extracts showed cytotoxic activity, while less than a quarter showed antimicrobial or anti-H1N1 activity. The typical aciduric fungus *Penicillium oxalicum* OUCMDZ-5207 showed similar growth but completely different chemical diversity at pH 3 and 7. The metabolites of OUCMDZ-5207 that were obtained only at pH 3 were identified as tetrahydroauroglaucin (**1**), flavoglaucin (**2**), and auroglaucin (**3**), among which auroglaucin showed strong selective inhibition of A549 cells with an IC_50_ value of 5.67 μM. These results suggest that acid stress can activate silent gene clusters to expand the diversity of secondary metabolites, and the bioprospecting of aciduric/acidophilic microorganism resources in Thai mangrove sediments may lead to the discovery of compounds with potential medicinal applications.

## Introduction

Microbes are important drug producers, however, it is becoming more difficult to obtain new drugs and drug leads from common environmental microorganisms, with less discovery of new microorganisms occurring and duplication of research on their metabolites. Over the last 10 years, we have been investigating microbes and their metabolites from extreme environments. Extreme microorganisms are those that live in a special environment, including places with high temperatures (>50°C), low temperatures (<20°C), high pressure (>35 MPa), high salinity (>3% NaCl), high pH (>pH 9), and low pH (<pH 4) (Wilson and Brimble, 2009), where ordinary microbes cannot survive. The different physiological characteristics of extreme microorganisms can create unique metabolic pathways and their secondary metabolites can have remarkable chemical diversity and interesting bioactivity (Wang et al., 2009, 2011a,b,c, 2013; Zheng et al., 2009, 2010, 2013; Peng et al., 2011a,b, 2018; Fan et al., 2013, 2018; Lin et al., 2015; Qin et al., 2016; Wang M. M. et al., 2017; Jin et al., 2018).

Acid-tolerant microbes are a type of extreme microorganisms, which include acidophilic and aciduric microbes. The optimum pH for the growth of acidophiles is <pH 3, while aciduric microorganisms can tolerate both extremely acidic and neutral conditions (Johnson, 1998). There have been few studies on acidophilic fungi resources. In 2019, Martina et al. summarized the taxonomically identified indigenous acidophilic fungi, but only nine species (*Acidea extrema*, A*cidiella bohemica*, *Acidiella uranophila*, *Acidomyces acidophilus*, *Acidomyces acidothermus*, *Acidothrix acidophila*, *Coniochaeta fodinicola*, *Neohortaea acidophila*, and *Soosiella minima*) were listed. All these fungi were obtained from highly acidic habitats, including highly acidic soil, rivers, hot springs, mines, drainage, and acidophilic algae (Hujslová et al., 2019). Furthermore, Berkeley Pit Lake in the United States and mangrove sediments in China are acid-tolerant fungal habitats (Wang Y. et al., 2017). Although there has been a lack of investigation regarding the diversity of acidophilic fungi, this microbiota has the potential for a variety of applications, including bioremediation technologies (Deshmukh et al., 2016). For instance, acidophilic fungi have been reported to play an important role in the passive remediation of acid mine drainage and the degradation of environmental pollutants, such as phenolic compounds and polymerized rubber. The metabolites of acidophilic fungi also have potential medicinal value. In 2017, the Stierle group published a review of their work on the bioactive metabolites isolated from the abandoned acid metal mine, Berkeley Pit Lake (Stierle et al., 2017). By 2017, new compounds and bioactive compounds occupied 65 and 68% of all the compounds from aciduric microorganisms, respectively, suggesting that acid-tolerant fungi are potential medicinal resources (Wang Y. et al., 2017).

Mangroves are a special marine plant community distributed in the intertidal zone of tropical and subtropical regions. The particular nature of the environment means that mangrove sediments possess strong acidity and have the characteristics of high nutrition, high salinity, and strong reducibility. Although there have been some reports regarding microorganisms from mangroves, there have been few investigations of acidophilic or aciduric microorganisms. In our previous work, some novel bioactive compounds were obtained from acid-tolerant fungi in the soil of the mangrove rhizosphere of the China Mangrove Nature Reserve (Fan et al., 2013; Lin et al., 2015; Qin et al., 2016; Fan et al., 2018; Jin et al., 2018). Examples include new indole-diterpenoids with Anti-influenza virus activity from *Penicillium camemberti* OUCMDZ-1492 (Fan et al., 2013) and new anthraquinone derivatives with Anti hepatitis B virus activity from *Penicillium* sp. OUCMDZ-4736 (Jin et al., 2018). These results suggest that further investigation of the aciduric fungi obtained from mangrove sediments and their bioactive secondary metabolites are worthy of attention.

Previously, most studies of microbes from Thai mangroves have focused on bacteria and yeast (Limtong et al., 2007, 2008; Am-In et al., 2008, 2011; Atipan et al., 2012; Songsumanus et al., 2013; Wongwongsee et al., 2013; Hwanhlem et al., 2014; H-Kittikun et al., 2015; Wanapaisan et al., 2018). Fungi from Thai mangroves, especially acid-tolerant fungi and their bioactive metabolites under conditions of acidic stress, have not previously been investigated. In this paper, we report acid-tolerant fungi and their bioactive metabolites obtained from Thailand mangrove sediments. Using morphologic observations, chemical diversity comparisons, and analysis of the phylogenetic relationships of genes including internal transcribed spacer (ITS)-ribosomal DNA (rDNA), β-tubulin, calmodulin, partial actin gene sequences, the acid-tolerant fungi were identified and classified, and the biodiversity and biological characteristics were evaluated. Furthermore, acid tolerance and antibiotic biological activity (antibacterial, cytotoxicity, and antiviral activity) were also studied. A typical aciduric fungus – *Penicillium sp.* OUCMDZ-5207 – was analyzed under acidic and neutral conditions, and the metabolites at pH 3 and their cytotoxicity were studied.

## Materials and Methods

### General Experimental Procedures

UV spectra were recorded on a Thermo Fisher Scientific NanoDrop One micro-spectrophotometer (Thermo Fisher Scientific, Waltham, MA, United States). ^1^H NMR, ^13^C NMR, and 2D NMR spectra were recorded on a Bruker Avance 500 MHz spectrometer with tetramethylsilane (TMS; Alfa Chemistry, United States) as an internal standard. Mass spectra were recorded on an Agilent 6200 Q-TOF MS system. TLC was performed on plates precoated with silica gel GF254 (10–40 μm, Qingdao Marine Chemical Ltd., Qingdao, China). Column chromatography was performed on silica gel (100–200, 200–300, and 300–400 mesh; Qingdao Marine Chemical Ltd., Qingdao, China), RP-18 gel (20–45 μm), and Sephadex LH-20 (Amersham Biosciences, United Kingdom). Medium-pressure liquid chromatography (MPLC; CXTH-3000, China) was performed using columns packed with RP-18 gel and an LC3000 system equipped with P3000A pump modules. Semipreparative high performance liquid chromatography (HPLC; Waters 1525, United States) was performed using a column (YMC-pakODS-A, 10 × 250 mm, 5 μm, 4 mL/min).

### Fungi Isolation

Fungi strains were isolated from the sediment around the roots of *Hibiscus tiliaceus*. Samples were collected from the mangrove habitat at PakMeng Beach, Thailand in October 2016. The samples were stored at –20°C and processed immediately for isolation and cultivation of the fungus after transport to the laboratory. The samples were then frozen at –20°C.

The gradient dilution method was used for fungi isolation. To remove superficial sediments and microbes, samples (1.0 g) were sterilized with 75% ethanol and rapidly washed three times with sterile water under sterile conditions. The samples were homogenized thoroughly and diluted to three different concentrations (0.1, 0.01, and 0.001) with sterile seawater. Then, 100-μL samples of each dilution were plated onto the corresponding isolation medium (Table 1) in triplicate. The acid medium was adjusted with citric acid and disodium hydrogen phosphate buffer solution to obtain the desired pH. The neutral medium was adjusted with NaOH. Chloramphenicol (100 μg/mL) was added to the medium to prevent the growth of bacteria during the incubation. The fungi were cultured at 28°C for at least 5 days. To ensure strain diversity, timely observations were conducted as much as possible. All the colonies were collected and purified on the plate repeatedly until a single colony was obtained, then inoculated onto slope media and stored at 4°C. Furthermore, these strains were also preserved in 20% glycerin liquid medium at −80°C.

**TABLE 1:** Ingredient of media used for isolation and fermentation of microbial strains ^a,b,c^.

Microbes	Medium	Ingredient
Fungi sample	SWS	1.0 g peptone, 10.0 g soluble starch,20.0 g agar
	PDA	200.0 g potato extract, 20.0 g dextrose, 20.0 g agar
	GYP	10.0 g glucose,1.0 g yeast extract, 2.0 g peptone;20.0g agar
	MYPG	3.0 g yeast extract, 3.0 g malt extract, 5.0 g peptone,20.0 g glucose,1.0 g K_2_HPO_4_3H_2_O, 0.50g MgSO_4_7H_2_O, 0.20 g FeCl_3_6H_2_O, 20.0 g agar
	Martin medium	10.0 g peptone, 10.0 g glucose, 1.0 g K_2_HPO_4_3H_2_O, 0.50 g MgSO_4_7H_2_O, 20.0 g agar
	Malt extract medium	30.0 g malt extract, 5.0 g tryptone, 20.0g agar
Pathogenic Microbes	Luria Bertani (LB)	5.0 g yeast extract, 10.0 g peptone, 5.0 g NaCl, 20.0 agar
	Zobell2116E	5.0 g peptone,3.0 g yeast extract, 20.0 g agar
	YPD	Peptone 2.0 g, yeast extract 1.0 g, glucose 2.0 g, 20.0 g agar

*^*a*^All mediums were prepared with nature sterile seawater and adjusted to appropriate pH before autoclaving at 121°C for 20 min. ^*b*^ The liquid media without agar were used for fermentation of fungi in bioassay and compound isolation. ^*c*^ The 2216E, YPD, and LB media were used to culture aquatic and human pathogenic microbial strains in the antimicrobial test.*

### DNA Extraction, PCR Amplification, and Sequencing

Single fungal colonies were transferred with a sterilized toothpick to 50 μL of a lysis buffer for PCR with a Direct PCR kit (Takara) in a sterilized microtube. The microtubes were placed at 80°C for pre-degeneration for 15 min and then centrifuged at low speed. A 2-μL sample of the supernatant was used as the template to amplify the fungal gene fragment in a polymerase chain reaction (PCR). internal transcribed spacer (ITS)-ribosomal DNA (rDNA) sequences were amplified with the primers ITS1F (5′-CTTGGTCATTTAGAGGAAGTAA-3′) (Gardes and Bruns, 1993) and ITS4 (5′-TCCTCCGCTTATTGATATGC-3′) (White et al., 1990). For the sequencing of partial β-tubulin gene, βt2a (5′-GGTAACCAAATCGGTGCTGCTTTC-3′) and βt2b (5′-ACCCTCAGTG TAGTGACC CTTGGC-3′) were used as the primers (Glass and Donaldson, 1995). Partial calmodulin gene was amplified using the primers cmd5 (5′-CCGAGTACAAGGAGGCCTTC-3′) and cmd6 (5′-CCGATAGAGGTCATAACGTG-3′) (O’ Donnell et al., 2000). For the sequencing of the partial actin gene, act-512F (5′-ATGTGCAAGGCCGGTTTCGC-3′) and ACT-783R (5′-TACGAGTCCTTCTGGCCCAT-3′) were used as the forward primer and reverse primer, respectively (Carbone and Kohn, 1999). The PCR reactions were performed in a final volume of 50 μL, which was composed of 2 μL of template DNA, 10 μL of 5 × PrimeSTAR Buffer (Mg2 + plus), 4 μL of dNTP Mix (2.5 mM of dATP, dCTP, dGTP and dTTP, respectively), 0.5 μL of forward primer (20 μM), 0.5 μL of reverse primer (20 μM), 0.5 μL of PrimeSTAR HS DNA Polymerase (2.5 U/μL), and 32.5 μL of ultrapure water. Amplification was carried out using the following thermal cycles: 30 cycles of 10 s at 98°C, 5 s at 55°C, and 60 s at 72°C. Then, the PCR products (5 μL) were loaded onto an agarose gel [1.2% agarose in 0.5 × TAE buffer and 5 μL of 1% ethidium bromide solution (m/v) per 100 mL of gel] and isolated using a gel extraction kit (E.Z.N.A., Omega, United States), according to the manufacturer’s protocol after electrophoresis at 100 V for 35 min. PCR products were submitted for sequencing (Shanghai Personalbio Biotechnology Co., Ltd., China).

### Phylogenetic Analysis

Fungal sequences were edited with Lasergene Software SeqMan (DNAStar Inc.). For preliminary identification, the sequences were compared with related sequences available in the National Center for Biotechnology Information (NCBI) to determine the identity of the sequence. All the fungal sequences were aligned using the CLC Sequence Viewer 6 software with the default parameters. All the identified ITS-rDNA sequences of the Thai mangrove fungi were submitted to NCBI GenBank and the accession numbers were obtained. All the sequences of β-tubulin, calmodulin, partial actin gene were shown in Supplementary Material. A phylogenetic tree was generated with the neighbor-joining (NJ) method in the MEGA 4 software and 1,000 replicates were used for the bootstrap analysis. iTOL online software was used to process the phylogenetic tree. The reference sequences showing the highest homology to the sequences from the Thai mangrove fungi were amplified.

### Preparation of Samples for Bioassay

The fungal strains were fermented with shaking at 28°C and 180 rpm for 7 day in a 500-mL conical flask containing 150 mL of Potato Dextrose Broth (PDB) liquid medium. The acid medium was prepared by adding citric acid and disodium hydrogen phosphate buffer solution. Three parallel lines were set for each experiment. The fermentation broth was extracted with an equal volume of ethyl acetate (EtOAc) three times. The dried extracts for bioassay were obtained by evaporation under reduced pressure.

### Isolation and Identification of Compounds

*Penicillium* sp. OUCMDZ-5207 was isolated from the sediment around the roots of the mangrove plant, *H. tiliaceus*, at pH 2.0. The strain was fermented under static conditions at 26°C for 35 day in twenty 1000-mL Erlenmeyer flasks each containing 300 mL of PDB liquid medium at pH 3.0. The fermentation broths were harvested and extracted three times with an equal volume of ethyl acetate (EtOAc). Removal of the solvent in vacuum gave 3.2 g of EtOAc extract. The EtOAc extracts were separated on a silica-gel column eluting with petroleum ether (PE)–EtOAc (v/v100:1, 50:1, 25:1, 10:1, 5:1, 1:1, 1:2, 1:5, and 1:10) to obtain nine subfractions (Fr.1–Fr.9). Fr.3 (80.1 mg) was further subjected to purification with an HPLC ODS column (v/v acetonitrile –H_2_O 7:3) to give Fr.3-1–Fr.3-4. Fr.3-4 (10.3 mg) was further purified using a HPLC Polymer Stabilized Cholesteric Texture column (v/v acetonitrile–H_2_O 13:20) to yield compounds 1 (3.21 mg, t_R_ 30 min) and 2 (4.32 mg, t_R_ 34 min). Fr.4 was further separated by Sephadex LH-20 column chromatography eluting with MeOH-CH_2_Cl_2_ (v/v 1:1) to give three subfractions (Fr.4-1–Fr.4-3). Fr.4-2 (5.6 mg) and subjected to silica gel column chromatography eluting with petroleum ether–EtOAc (v/v 35:1, 25:1, and 10:1) to give compound 3 (2.67 mg).

Tetrahydroauroglaucin (**1**): Light yellow amorphous solid. C_19_H_26_O_3_. UV(MeOH) λ_max_ (logε) 240 (0.17), 275 (0.08), 392 (0.01) nm; ^1^H-NMR (DMSO-*d*_6_, 600 MHz) δ: 0.83 (3H, t, *J* = 6.7 Hz, H-7′), 1.26 (4H, m, H-5′, H-6′), 1.41 (2H, quintet, *J* = 7.3 Hz, H-4′), 2.62 (3H, s, H-4″), 2.19 (3H, s, H-5″), 2.46 (2H, q, *J* = 1.7 Hz, H-3), 3.16 (2H, d, *J* = 7.4 Hz, H-1″), 9.16 (1H, brs, 5-OH), 5.20 (1H, t, H-2″), 6.51 (1H, m, H-2′), 6.94 (1H, s, H-4), 5.78 (1H, dt, *J* = 15.9, H-1′), 9.99 (1H, s, 1-CHO), 11.60 (1H, s, 2-OH). ^13^C-NMR (DMSO-*d*_6_, 125 MHz) δ 197.3 (1-CHO), 152.9 (C-2), 146.9 (C-5), 140.5 (C-2′), 132.7 (C-3″), 127.7 (C-3), 124.7 (C-4), 125.7 (C-6), 121.6 (C-2″), 121.0 (C-1′), 117.4 (C-1), 33.2 (C-3′), 30.9 (C-5′), 28.4 (C-4′), 26.9 (C-1″), 25.6 (C-5″), 22.0 (C-6′), 17.7 (C-4″), 14.0 (C-7′). ESI-MS *m/z* 301[M-H]^–^.

Flavoglaucin (**2**): Light yellow amorphous solid. C_19_H_28_O_3_. UV(MeOH) λ_max_ (logε) 214 (1.33), 255 (0.99), 300 (0.76) nm; ^1^H-NMR (DMSO-*d*_6_, 600 MHz) δ: 11.68 (1H, s, 2-OH), 10.19 (1H, s, 7-CHO), 6.92 (1H, s, H-4), 5.18 (1H, d, *J* = 14.8 Hz, H-2″), 8.95 (1H, brs, 5-OH), 3.13 (2H, d, *J* = 7.4 Hz, H-1″), 2.80 (2H, t, *J* = 7.7 Hz, H-1′), 1.65 (3H, s, H-5″), 1.61 (3H, s, H-4″), 1.40 (2H, m, H-2′), 1.19–1.23 (8H, m, H-3′, 4′, 5′, 6′), 0.80 (3H, t, *J* = 6.7 Hz, H-7′) ^13^C NMR (DMSO-*d*_6_, 125 MHz) δ 197.2 (1-CHO), 153.2 (C-2), 146.9 (C-5), 132.5 (C-3″), 129.0 (C-3), 126.8 (C-6), 125.2 (C-4), 121.8 (C-2″), 117.6 (C-1), 31.3 (C-2′), 31.5 (C-4′), 29.0 (C-5′), 28.6 (C-3′), 26.7 (C-1″), 25.6 (C-5″), 23.2 (C-1′), 22.1 (C-6′), 17.7 (C-4″), 14.0 (C-7′). ESI-MS *m/z* 303[M-H]^–^.

Auroglaucin (**3**): Yellow amorphous solid. C_19_H_22_O_3_. UV(MeOH) λ_max_ (logε) 230 (1.47), 211 (1.22), 278 (1.13) nm. ^1^H-NMR (DMSO-*d*_6_, 600 MHz) δ: 11.71 (1H, s, 2-OH), 10.03 (1H, s, 7-CHO), 6.95 (1H, s, H-4), 5.18 (1H, t, *J* = 14.8 Hz, H-2″), 9.43 (1H, brs, 5-OH), 3.17 (2H, d, *J* = 7.3 Hz, H-1″), 6.82 (1H, d, *J* = 15.6 Hz, H-1′), 6.15 (1H, m, H-5′), 1.60 (3H, s, H-4″), 6.60 (1H, m, H-2′), 6.32 (2H, m, H-3′, 4′), 5.74 (1H, m, H-6′), 1.71 (3H, d, *J* = 6.8 Hz, H-7′); ^13^C NMR (DMSO-*d*_6_, 125 MHz) δ 196.9 (1-CHO), 153.3 (C-2), 147.7 (C-5), 131.2 (C-3″), 134.5 (C-3), 121.4 (C-6), 124.9 (C-4), 123.0 (C-2″), 116.9 (C-1), 124.5 (C-2′), 131.9 (C-4′), 128.4 (C-5′), 132.8 (C-3′), 26.9 (C-1″), 17.7 (C-5″), 130.9 (C-1′), 137.8 (C-6′), 25.6 (C-4″), 18.3 (C-7′). ESI-MS *m/z* 297[M-H]^–^.

### Anti-microbial Assay

The paper disk-agar diffusion method (Tamura et al., 1968) was used to evaluate the inhibition of pathogenic microorganisms, including nine aquatic pathogens (*Edwardsiella tarda*, *Vibrio parahaemolyticus*, *Vibrio Vulnificus*, *Vibrio alginolyticus*, *Aeromonas hydrophila*, *Pseudoalteromonas nigrifaciens*, *Shewanella marisflavi*, *Vibrio splendidus*, and *Bacillus cereus*) and eight human pathogens (*Staphylococcus aureus*, *Staphylococcus aureus* Rosenbach, *Escherichia coli*, *Bacillus subtilis*, *Clostridium perfringens*, *Candida albicans*, *Candida glabrata*, and *Pseudomonas aeruginosa*). The initial concentration of the EtOAc extract was 1 mg/mL. Ciprofloxacin and ketoconazole at concentrations of 0.1 mg/mL were used as positive controls. The pathogenic microorganisms were cultivated in a 50-mL centrifuge tube containing 30 mL of liquid medium and shaken at 28°C and 180 rpm for 12 h. LB medium was used for human pathogenic bacteria, 2116E medium for aquatic pathogenic bacteria, and YPD medium for human pathogenic fungi. The pathogenic strains were coated on a corresponding plate and incubated at 37°C for 30 min. Paper discs with 10 μL of the sample or positive controls (three parallels) were volatilized to dryness and placed onto an agar plate coated with the bacterial suspension. The plates were incubated for 12 h at 37°C. The negative control was methanol. Then, the diameters of the inhibition zones were recorded as the observed antibacterial activity.

### Cytotoxic Assay

The cytotoxicity against human breast adenocarcinoma cell line MCF-7 and human lung adenocarcinoma cell line A549 was evaluated by the MTT method (Mosmann, 1983). The EtOAc extracts, compounds, and positive control (adriamycin) were dissolved in dimethyl sulfoxide (DMSO), DMSO was a negative control. The cell lines were cultivated in RPMI-1640 medium with 10% FBS under a humidified atmosphere with 5% CO_2_ at 37°C. Cell suspensions of MCF-7 and A549 (100 μL) were inoculated into 96-well plates (5 × 10^3^ cells/well) and incubated under the above conditions for 24 h. Then, 100-μL samples with final concentrations of 100 μg/mL of extract, 10 μM of a compound, and 1 μM of adriamycin were added to each well in triplicate and the culture mediums were incubated for 72 h. The RPMI-1640 medium containing 5% FBS was used as a blank control. MTT (methylthiazolyldiphenyl-tetrazolium bromide) solution (20 μL; 5 mg/mL in FBS) was added into each well and the wells were incubated for 4 h at 37°C. The medium containing MTT was gently removed with a pipette and 150 μL of DMSO was added to each well to dissolve the formed formazan crystals. The absorbance was recorded on a Spectra Max Plus plate reader at 570 nm. The inhibition rate was calculated as [(OD blank - OD sample)/OD blank] × 100%.

### Anti-H1N1 Assay

The cytotoxicity of the samples against MDCK cells was evaluated by the method described above. Non-toxic samples (inhibition rates <0) were evaluated for antiviral bioactivity against the influenza virus H1N1 [A/Puerto Rico/8/34 (H1N1), PR/8] by the MTT method. The Madin-Darby Canine Kidney (MDCK) cells were digested by trypsin as monolayer cells and incubated with the influenza virus H1N1 at 37°C for 1 h. Then, the culture medium was discarded and a new RPMI 1640 medium with 20 μL samples 50 μg/mL were added. The inhibition was evaluated by the MTT method the same as for cytotoxicity, as described above.

## Results and Discussion

### Isolation and Phylogenetic Diversity of Aciduric Fungi Associated With Thai Mangrove Sediment

**FIGURE 1:**
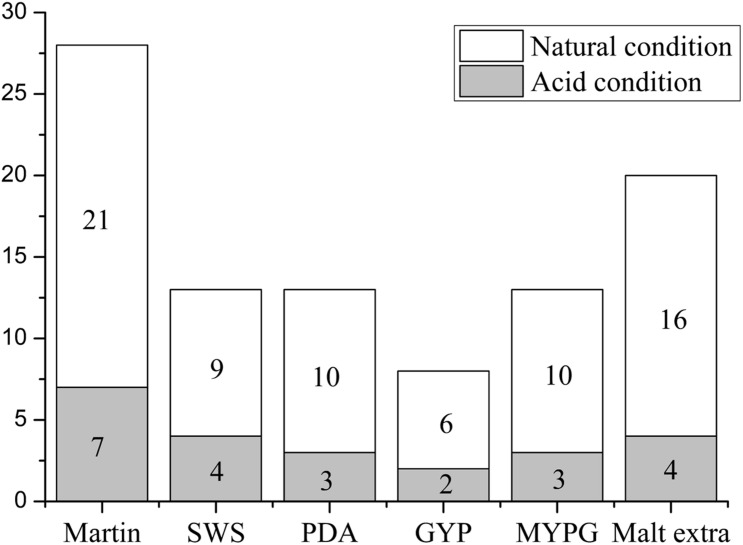
Numbers of fungi isolated from Thai mangrove samples using different media under acidic (pH 3.0) and natural (pH 5.0) conditions (95 fungi strains, 72 at pH 5.0 and 23 at pH 3.0). The horizontal axis represents the type of culture medium (SWS: peptone starch agar; PDA: potato dextrose agar; GYP: glucose yeast extract peptone; and MYPG: malt extract yeast extract peptone glucose), and the vertical axis represents the number of fungi isolates.

A total of 237 fungi strains were isolated from the acidic sediment collected from around the roots of *H. tiliaceus*, a mangrove plant from PakMeng Beach, Thailand. Six types of culture medium (Table 1) at two different pH values were used for isolation of the fungi. The acidity of the sediment sample was pH 5.0 and this pH value was used for the isolation. Additionally, pH 3.0 was used for the isolation to increase the possibility of obtaining acidophilic microbes. In total, 163 and 74 fungal strains were obtained under natural (pH 5.0) and acidic (pH 3.0) conditions, respectively. Duplicates were removed by detailed morphological observation, pigment formation, TLC characteristics, and HPLC fingerprint analysis of the crude extracts of the metabolites. After this assessment, there remained 95 individual fungi strains (72 at pH 5.0 and 23 at pH 3.0). These mangrove fungi grew better under natural conditions (pH 5.0) compared with the highly acidic environment (pH 3.0) (Figure 1). Compared with the other mediums used for isolation, the Martin medium was found to be more suitable for fungi growth, which may be related to the presence of more inorganic salts in this medium. The fungi may grow better in a medium rich in inorganic salts because of the characteristics of the natural fungal environment where the mangrove sediments are repeatedly washed by seawater and that deposits a large amount of inorganic salts.

We investigated research references on fungi derived from the semi-mangrove plant *H. tiliaceus* from 2008 to 2020 (Li et al., 2008, 2010; Yan et al., 2010, 2012; Wang et al., 2012). Most results discussed the plant endophyte and their metabolites. The fungal genera included *Aspergillus flavus*, *Penicillium commune*, and *Eurotium rubrum*, among which *A. flavus* strain was isolated from the roots, and the others were isolated from flowers or stems. There is no reference reported on pH adaptability of these fungi from the soil around *H. tiliaceus* roots. So it is the first time to report the characteristics of acid-tolerant rhizosphere fungi derived from *H. tiliaceus* of Thailand mangroves. Mangrove soil is a special acidic geological environment, but there have been few reports of acid-tolerant fungi from this environment. Generally, a highly acidic environment is not conducive to microbial growth; however, our results showed more than a quarter of the fungi (23/95) showed better growth in the highly acidic medium (pH 3.0), which indicated that the microbial community in mangrove sediments associated with *H. tiliaceus* in Thailand has distinctive acidophilic properties.

**FIGURE 2:**
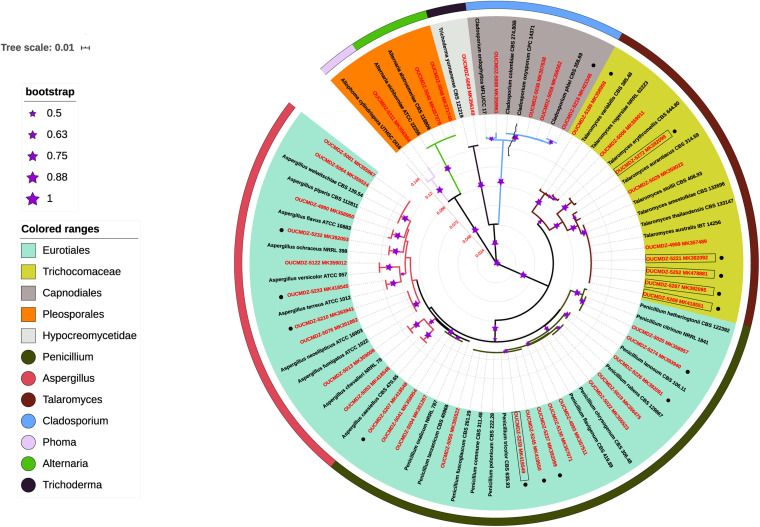
Phylogenetic relationship of 41 representative fungal isolates based on ITS rDNA gene sequences using the neighbor-joining method. The scale bar represents 0.01 substitutions per nucleotide. Different orders and genera are represented by different colors. The size of the asterisk indicates different bootstrap values from 1000 replicates (a larger asterisk means a higher credibility). The red label indicates the strains isolated from the experiment, and the black label indicates the reference strains in the database. Acidophilic fungi are represented by boxes. The strains obtained under pH 3.0 are indicated by black spots.

To investigate the phylogenetic relationship of these mangrove fungi, the ITS sequences were compared using Clustal X software, the isolates with duplicated ITS sequences were excluded, and the remaining 41 strains were analyzed for phylogenetic relationships. According to the blast results compared with the sequences already registered in the NCBI gene databank, all the isolated fungi belonged to the Ascomycota phylum. These fungi showed 96–100% similarity to their closest relatives (Table 2). Based on the analysis of homology of the ITS rDNA gene, a phylogenetic relationship containing 41 representative fungi isolates was constructed by the neighbor-joining method (Figure 2). Considering the fact that the ITS phylogenetic analysis was not sufficient, different gene combinations were used for different genera. The β-tubulin gene was used for 33 isolates identified as *Penicillium*, *Talaromyces*, *Aspergillus* with the ITS genetic marker (Supplementary Figure 2). The phylogenetic relationship based on β-tubulin genes showed OUCMDZ-5245 and 5259 were clustered together with *Talaromyces* which were different from the ITS result *Penicillium*. Calmodulin and actin genes were used to analyze strain 5245 and 5259, respectively, and the results also showed that they all belonged to *Talaromyces*. At the level of genetic analysis, these two strains were considered more reliable as *Talaromyces*. Calmodulin and actin genes were used for analyzing OUCMDZ-5058, 5216, 5036, 5008, 5111 and 5083, 5040, 5048, respectively (Supplementary Figures 3, 4). Except for strain 5111, all the identification results were corresponded to the ITS rDNA analyzing results. The actin gene was used for identification of 5111, and the result showed that 5111 belonged to *Allophoma* which was the same as the result of ITS. Combined with all the genetic analysis results, all the identified fungi belonged to one phylum (Ascomycota), three classes (Eurotiomycetes, Dothideomycetes, and Sordariomycetes), four orders (Eurotiales, Capnodiales, Pleosporales, and Hypocreomycetidae) and seven genera including *Penicillium* (12 isolates), *Aspergillus* (10 isolates), *Talaromyces* (11), *Cladosporium* (4), *Alternaria* (2), *Allophoma* (1), and *Trichoderma* (1). Among them, 80.5% of the strains belonged to the order Eurotiales. *Penicillium*, *Talaromyces*, and *Aspergillus* were the dominant genera in this mangrove ecosystem and were extensively distributed.

**TABLE 2:** Phylogenetic affiliations and the classification of 41 representative acid-tolerant fungal isolates from Thai mangrove samples.

Isolate No.	Class	Order	Family	Genus	Accession No.	Closest identified relative.	Identity (%)	Numbers
OUCMDZ-5020	Eurotiomycetes	Eurotiales	Aspergillaceae	*Penicillium*	MK358957	*Penicillium citrinum* NRRL 1841 (NR_121224.1)	99	12
OUCMDZ-5041					MK358954	*Penicillium oxalicum* NRRL 787 (NR_121232.1)	99	
OUCMDZ-5050					MK355522	*Penicillium polonicum* CBS 222.28 (NR_103687.1)	100	
OUCMDZ-5130					MK357071	*Penicillium chrysogenum* CBS 306.48 (NR_077145.1)	99	
OUCMDZ-5019					MK356475	*Penicillium rubens* CBS 129667 (NR_111815.1	99	
OUCMDZ-5207^a^					MK418548	*Penicillium tanzanicum* CBS 140968 (NR_158820.1)	99	
OUCMDZ-5226^a^					MK392091	*Penicillium lanosum* CBS 106.11 (NR_163539.1)	99	
OUCMDZ-5274^a^					MK393940	*Penicillium hetheringtonii* CBS 122392 (NR_111482.1)	99	
OUCMDZ-5004					MK351267	*Penicillium oxalicum* NRRL 787 (NR_121232.1)	100	
OUCMDZ-5237^a^					MK392099	*Penicillium commune* CBS 311.48 (NR_111143.1)	99	
OUCMDZ-4993					MK357511	*Penicillium flavigenum* CBS 419.89 (NR_103695.1)	100	
OUCMDZ-5022					MK355523	*Penicillium rubens* CBS 129667 (NR_111815.1)	100	
OUCMDZ-5122				*Aspergillus*	MK359012	*Aspergillus versicolor ATCC 957* (NR_131277.1)	99	10
OUCMDZ-5013					MK359008	*Aspergillus chevalieri NRRL 78* (NR_135340.1)	100	
OUCMDZ-5053					MK358986	*Aspergillus caesiellus CBS 470.65* (NR_077146.1)	99	
OUCMDZ-4990					MK358960	*Aspergillus flavus ATCC 16883* (NR_111041.1)	99	
OUCMDZ-5233^a^					MK418545	*Aspergillus terreus* ATCC 1012 (NR_131276.1)	99	
OUCMDZ-5064					MK355524	*Aspergillus welwitschiae* CBS 139.54 (NR_137513.1)	100	
OUCMDZ-5081					MK355967	*Aspergillus piperis* CBS 112811 (NR_077191.1)	99	
OUCMDZ-5232^a^					MK392093	*Aspergillus ochraceus* NRRL 398 (NR_077150.1)	100	
OUCMDZ-5076					MK351862	*Aspergillus ellipticus* ATCC 16903 (NR_138337.1)	100	
OUCMDZ-5210^a^					MK393941	*Aspergillus fumigatus* ATCC 1022 (NR_121481.1)	100	
OUCMDZ-5029			Trichocomaceae	*Talaromyces*	MK359023	*Talaromyces aurantiacus* CBS 314.59 (NR_103681.2)	97	11
OUCMDZ-5006					MK359011	*Talaromyces rogersiae* NRRL 62223 (NR_155914.1)	99	
OUCMDZ-5185^a^					MK358958	*Talaromyces variabilis* CBS 385.48 (NR_103670.2)	96	
OUCMDZ-5259^ab^						*Talaromyces liani* NRRL 1009 (NR_077206.1)	99	
OUCMDZ-5245^ab^						*Talaromyces minioluteus* CBS 642.68 (MN969409.1)	83	
OUCMDZ-4999					MK357489	*Talaromyces amestolkiae* CBS 132696 (NR_120179.1)	99	
OUCMDZ-5267^a^					MK392095	*Talaromyces siamensis* CBS 475.88 (NR_103683.2)	99	
OUCMDZ-5269^a^					MK418551	*Talaromyces australis* IBT 14256 (NR_147431.1)	99	
OUCMDZ-5221^a^					MK392092	*Talaromyces stollii* CBS 408.93 (NR_111781.1)	99	
OUCMDZ-5272^a^					MK392098	*Talaromyces erythromellis* CBS 644.80 (NR_121531.1)	96	
OUCMDZ-5252^a^					MK478881	*Talaromyces thailandensis* CBS 133147 (NR_147428.1)	99	
OUCMDZ-5058	Dothideomycetes	Capnodiales	Cladosporiaceae	*Cladosporium*	MK356562	*Cladosporium colombiae* CBS 274.80B (NR_119729.1)	99	4
OUCMDZ-5216^a^					MK421546	*Cladosporium phlei* CBS 358.69 (NR_120013.1)	100	
OUCMDZ-5036					MK357638	*Cladosporium oxysporum* CPC 14371 (NR_152267.1)	100	
OUCMDZ-5008					MK358961	*Cladosporium endophytica* MFLUCC 17 (NR_158360.1)	98	
OUCMDZ-5111		Pleosporales	Pleosporineae	*Allophoma*	MK356384	*Allophoma cylindrispora* UTHSC DI16 (NR_158276.1)	98	1
OUCMDZ-5040				*Alternaria*	MK357070	*Alternaria alstroemeriae* CBS 118809 (NR_163686.1)	99	2
OUCMDZ-5048					MK371740	*Alternaria eichhorniae* ATCC 22255 (NR_111832.1)	99	
OUCMDZ-5083	Sordariomycetes	Hypocreomycetidae	Hypocreaceae	*Trichoderma*	MK356149	*Trichoderma yunnanense* CBS 121219 (NR_134419.1)	99	1
**Total Numbers**	**3**	**4**	**5**	**7**				**41**

*^*a*^ The strains were isolated at pH 3. Other strains were isolated at pH 5. ^*b*^ Phylogenetic affiliations were constructed based on ITS rDNA genes except for OUCMDZ-5259 and 5254 which were based on β-tubulin genes shown in Supplementary Material.*

**FIGURE 3:**
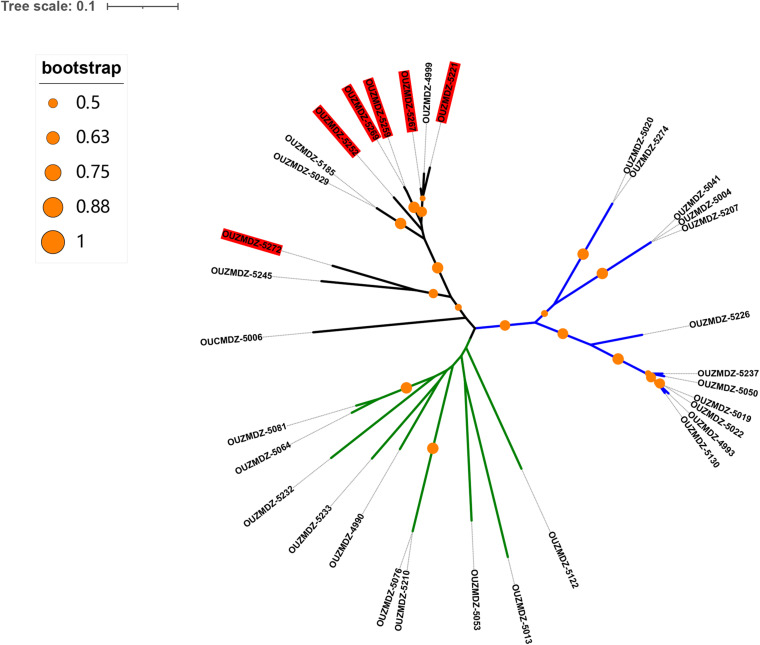
The unrooted phylogenetic tree constructed using all isolates based on β-tubulin gene sequences. The red colored background indicates acidophilic fungi. The blue, green, and black branches represent the genus *Penicillium*, *Aspergillus*, and *Talaromyces*, respectively. It can be seen that all the acidophilic isolates belonged to the genus *Talaromyces*.

To understand the acid tolerance of these fungi, all the 41 strains obtained under (pH 3.0 and pH 5.0) were incubated under extremely acidic conditions (pH 2.0) and neutral conditions (pH 7), and the growth was observed. The results indicated that all of the 41 fungi strains could grow at pH 5.0, six (OUCMDZ-5221, 5252, 5259, 5267, 5269, and 5272) could not survive at pH 7.0 but grew well at pH 2.0, and two (OUCMDZ-4999 and 5111) could not survive at pH 2.0 or 3.0. The six isolates (OUCMDZ-5221, 5252, 5259, 5267, 5269, and 5272) can be characterized as acidophilic fungi that cannot survive in a neutral environment and have optimal growth at pH values between 2 and 5, and interestingly, all these six isolates belonged to the genus *Talaromyces*. These results came from the unrooted phylogenetic tree constructed with β-tubulin gene sequences (Figure 3).

**FIGURE 4:**
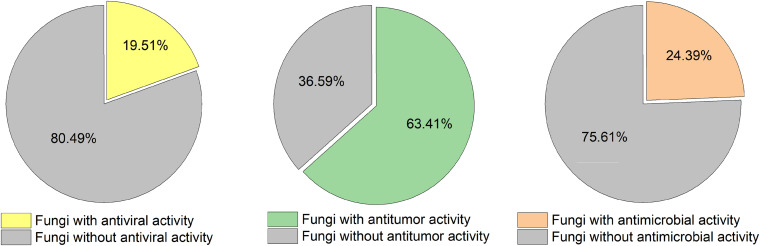
Percentage of bioactive acid-tolerant fungal strains isolated from Thai mangrove sediment.

All six acidophilic fungi were clustered in the branch of the genus *Talaromyces*. We summarized all the references on isolation and identification of *Talaromyces* since 2015 and found that no acidic culture conditions were involved in the isolation and culture process (including 34 new species of *Talaromyces*) (Adhikari et al., 2015; Visagie et al., 2015; Chen et al., 2016; Luo et al., 2016; Yilmaz et al., 2016a,b; Barbosa et al., 2018; Jiang et al., 2018; Rajeshkumar et al., 2019). However, in our experiments, all six acidophilic fungi isolates belonged to the genus *Talaromyces*. It was almost impossible to isolate these strains under neutral conditions. Although it was difficult to conclude whether the acidophilic fungi *Talaromyces* isolated by us were new species or not, these results revealed the acidophilic properties of the genus *Talaromyces* for the first time. This indicates that more samples of *Talaromyces* could be separated under acidic conditions.

The secondary metabolites of this genus have shown great potential in their chemical and bioactive diversity. Terpenoids, alkaloids, and polyketides with multiple bioactivities (including antibiotic, cytotoxic, and anti-HBV activity) have been reported as metabolites from *Talaromyces* (Hayashi et al., 2012; Kumar et al., 2013; Wu et al., 2015; Zhai et al., 2015; Fu et al., 2016; Guo et al., 2016; Xie et al., 2016; Zhao et al., 2016, 2019; Zhi et al., 2016; Cai et al., 2017; Küppers et al., 2017; Chen et al., 2018; Wang et al., 2018), with another review paper published in 2020 (Lan and Wu, 2020). Our investigation provides good indications for future studies on the metabolites of this genus, which require further exploration. The mangrove sediments in Thailand are the best source of acid-tolerant microbes for investigating the acidophilic properties and diverse metabolites of the genus *Talaromyces*.

### Bioactive Diversity of Aciduric Fungi Derived From Thai Mangrove Sediment

Forty-one representative fungi were fermented in a PDB medium at pH 3.0 and/or pH 7.0 based on the results of the growth of the isolates in the acid resistance assessment (detailed in Supplementary Table 1). All of the EtOAc extracts were tested for antimicrobial activity against nine aquatic and eight human pathogenic microbes. Nearly a quarter of the samples showed inhibitory effects (Table 3). The fungal strain OUCMDZ-5130 displayed broad-spectrum inhibitory effects against *C. albicans*, *C. glabrata*, *E. coli*, *V. alginolyticus*, *P. nigrifaciens*, *V. splendidus*, *S. aureus subsp. aureus* Rosenbach, *E. tarda*, and *B. cereus*. The strains OUCMDZ-5081, 5210, 5233, and 5245 showed weak antibacterial activity against the aquatic pathogens *P. nigrifaciens* and *V. alginolyticus*, while the strains OUCMDZ-4993, 5020, and 5040 showed moderate antibacterial activity against the aquatic pathogen *B. cereus*. These fungi inhibited more aquatic pathogens than human pathogens.

**TABLE 3:** The antimicrobial activity of acid-tolerant fungal EtOAc extracts at 1000 μg/mL ^*a,b*^.

Strain No.	*C. albicans*	*C. glabrata*	*E. coli*	*V. alginolyticus^*d*^*	*P. nigrifaciens^*d*^*	*V. splendidus^*d*^*	*S. aureus*	*E. tarda^*d*^*	*B. cereus^*d*^*
OUCMDZ-5130	+	+	+++	++	+	−	++	++	+
OUCMDZ-5081	−	−	−	+	+	−	−	−	−
OUCMDZ-5210^*c*^	−	−	−	+	+	−	−	−	−
OUCMDZ-5233^*c*^	−	−	−	+	+	−	−	−	−
OUCMDZ-5245^*c*^	−	−	−	+	+	−	−	−	−
OUCMDZ-5048	−	−	−	−	+	−	−	−	−
OUCMDZ-5064	−	−	−	−	+	−	−	−	−
OUCMDZ-5040	−	−	−	−	−	+	−	−	+++
OUCMDZ-5020	−	−	−	−	+	−	−	−	++
OUCMDZ-4993	−	−	+	−	−	+	−	−	++

*^*a*^ +++strong: inhibition zone diameter (IZD) ≥18 mm; ++moderate: IZD = 10–17 mm; + weak: IZD < 10 mm; − non-active: IZD = 0 mm. ^*b*^ IDZs of ciprofloxacin (positive control of bacteria) at 100 μg/mL were 18, 20, 21, 18, 21, 20, and 17 mm for *E. coli*, *V. alginolyticus*, *P. nigrifaciens*, *V. splendidus*, *S. aureus* Rosenbach, *E. tarda*, *B. cereus*, respectively. IDZs of ketoconazole (positive control of *C. albicans* and *C. glabrata*) at 100 μg/mL were 19, 22mm. ^*c*^ The fungal strains were fermented in PDA liquid medium at pH 3. Other fungal strains were fermented at pH 7. ^*d*^ Aquatic pathogens, the others were human pathogens.*

The extracts were also evaluated for cytotoxicity against A549 and MCF-7 tumor cell lines at a concentration of 100 μg/mL (Table 4). The positive control adriamycin (1 μM) showed 80 and 72% inhibition of A549 and MCF-7 cells, respectively. The strains, which produced extracts that showed more than 50% inhibition, were defined as active strains. The results showed that 26 of the fungal strains (63.4%) were active, 13 of which had been fermented at pH 3. Eight strains, OUCMDZ-5013, 5041, 5076, 5083, 5221, 5237, 5245, and 5274, showed more than 60% inhibition of both A549 and MCF-7 cell lines. The strains OUCMDZ-5048 and 5207 only showed inhibitory activity against A549 cells with 92 and 53% inhibition, respectively. The strains OUCMDZ-5053 and 5008 stimulated the proliferation of A549 cells and inhibited the growth of MCF-7 cells.

**TABLE 4:** Cytotoxicity of acid-tolerant fungal EtOAc extracts at 100 μg/mL.

Strain No.	A549(%)	MCF-7(%)	Strain No.	A549(%)	MCF-7(%)
OUCMDZ-4993	34%0.98	31%1.09	OUCMDZ-5083	95%1.43	75%1.35
OUCMDZ-4990	83%0.78	42%1.02	OUCMDZ-5111	36%0.65	17%0.86
OUCMDZ-4999	45%1.78	4%0.36	OUCMDZ-5122	46%1.23	4%1.20
OUCMDZ-5004	84%1.97	52%1.05	OUCMDZ-5130	10%0.23	30%0.68
OUCMDZ-5006	61%1.57	29%0.79	OUCMDZ-5185^a^	47%1.03	43%1.09
OUCMDZ-5008	−105%1.45	42%0.79	OUCMDZ-5207^a^	53%1.98	−68%0.67
OUCMDZ-5013	71%0.98	76%0.86	OUCMDZ-5210^a^	27%0.78	46%0.32
OUCMDZ-5019	47%1.62	22%1.89	OUCMDZ-5216^a^	58%0.67	44%0.45
OUCMDZ-5020	45%0.56	37%1.79	OUCMDZ-5221^a^	87%0.97	80%0.78
OUCMDZ5022	65%1.79	1%1.90	OUCMDZ-5226^a^	84%0.84	31%0.45
OUCMDZ-5029	18%1.45	19%0.78	OUCMDZ-5232^a^	85%0.78	54%1.09
OUCMDZ-5036	21%1.27	21%1.67	OUCMDZ-5233^a^	91%1.65	53%0.12
OUCMDZ-5040	40%1.68	53%1.07	OUCMDZ-5237^a^	86%1.79	82%0.95
OUCMDZ-5041	83%0.93	62%0.54	OUCMDZ-5245^a^	86%1.45	67%1.35
OUCMDZ-5048	92%0.79	−140%0.56	OUCMDZ-5252^a^	43%0.12	23%0.45
OUCMDZ-5050	54%0.98	51%0.87	OUCMDZ-5259^a^	88%0.67	57%0.45
OUCMDZ-5053	−243%1.67	25%0.56	OUCMDZ-5267^a^	55%0.69	44%0.36
OUCMDZ-5058	40%0.93	16%0.98	OUCMDZ-5269^a^	71%1.09	21%0.98
OUCMDZ-5064	87%1.23	47%1.05	OUCMDZ-5272^a^	33%1.08	54%1.23
OUCMDZ-5076	76%0.78	59%0.97	OUCMDZ-5274^a^	80%1.98	59%1.45
OUCMDZ-5081	82%0.67	24%0.67			

*Each datum represents the average inhibition and the standard deviation of three independent experiments. ^*a*^ The fungal strains were fermented in PDA liquid medium at pH 3. Other fungal strains were fermented at pH 7.*

Extracts of 41 fungi were evaluated for cytotoxicity against MDCK cell lines and the results showed that 28 strains (68.2%) were not cytotoxic at a concentration of 50 μg/mL. These non-cytotoxic strains were then selected to test for anti-H1N1 virus activity at the same concentration (Table 5). Eight strains, OUCMDZ-5008, 5013, 5022, 5029, 5040, 5048, 5083, and 5111, showed anti-H1N1 virus activity, among which OUCMDZ-5040 was the most active against the H1N1 virus with 42.1% inhibition, while the positive control, ribavirin, showed 47.0% inhibition at 50 μg/mL. Interestingly, all the strains with anti-H1N1 virus activity were fermented under neutral conditions (pH 7.0), which was different from the results relating to the anti-tumor activity (where 50.0% of the strains were fermented at pH 3.0).

**TABLE 5:** Anti-H1N1 activities of acid-tolerant fungal EtOAc extracts at 50 μg/ml ^*a*^.

Strain No.	Inhibition (%)	Strain No.	Inhibition (%)
OUCMDZ-5008	25% ± 0.32	OUCMDZ-5040	42% ± 1.07
OUCMDZ-5013	17% ± 1.45	OUCMDZ-5048	23% ± 0.69
OUCMDZ-5022	14% ± 0.67	OUCMDZ-5083	12% ± 1.09
OUCMDZ-5029	14% ± 0.98	OUCMDZ-5111	20% ± 1.56

*The cytotoxicity of the sample against the MDCK cell was less than zero percent. Each datum represents the average inhibition and standard deviation of three independent experiments. The strains with an inhibition rate of less than 5% were considered to be inactive and are not presented in the table. ^*a*^ The fungal strains were fermented in PDA liquid medium at pH 7.*

The antimicrobial, cytotoxicity and antiviral activities of all the isolated aciduric and acidophilic fungal metabolites were evaluated. Nearly three-quarters of the fermentation extracts (26/41) showed cytotoxicity against A549 or/and MCF-7 cells, while less than a quarter of the isolates showed antimicrobial (10/41) or anti-H1N1 virus (8/41) activity (Figure 4). Most of the cytotoxic isolates were fermented under acidic conditions (pH 3), while all the isolates with anti-H1N1 virus activity were fermented under neutral conditions (pH 7.0). The metabolites of these acid-tolerant fungi did not show obvious antibacterial and antiviral activities but showed outstanding cytotoxic activities. These results suggest that an extremely acidic environment was not conducive to the growth of common microorganisms. However, these extreme conditions did not affect the survival of the acid-tolerant fungi, leading to the production of cytotoxic metabolites through population competition. These cytotoxic substances could potentially help mangrove plants to resist being eaten by other animals. More strains had inhibitory activity against aquatic pathogens than against human pathogens, which may be because the fungi exist in the rhizosphere environment of mangrove plants and protect the mangroves against pathogens.

### The Metabolites From *Penicillium* sp. OUCMDZ-5207 at pH 3

**FIGURE 5:**
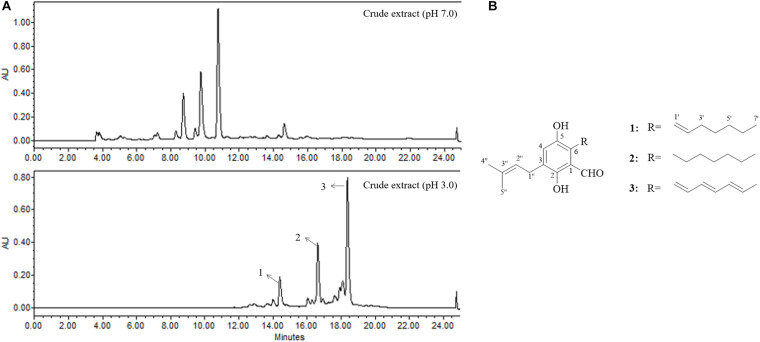
**(A)** The HPLC profile of secondary metabolites from OUCMDZ-5207 at pH 7.0 and pH 3.0 (HPLC gradient elution: 0–25 min, 10–100% CH_3_OH; UV wavelength: 300 nm; flow rate: 1 mL/min). **(B)** The chemical structures of compounds **1**–**3**.

The fungus strain OUCMDZ-5207 was identified as *Penicillium* according to the morphology and ITS sequences (Accession No. MK418548). No significant differences were observed in the growth or spore germination of OUCMDZ-5207 cultured at pH 3 and pH 7, but differences were found in the chemical diversity and the biomass under the acidic and neutral conditions (Figure 5). The EtOAc extracts fermented at pH 3 showed selective inhibitory activity against A549 cells with a 53% inhibition rate compared with a weak proliferative effect on MCF-7 cells. The metabolite yield at pH 3.0 (3.1240 g/L) far exceeded that at pH 7.0 (0.1236 g/L). Furthermore, compounds with retention times from 14 to 20 min were only produced at pH 3.0. Thus, strain OUCMDZ-5207 fermented at pH 3.0 was selected for chemical study, which resulted in the isolation and identification of three compounds that were only produced at pH 3.0, tetrahydroauroglaucin (**1**), flavoglaucin (**2**), and auroglaucin (**3**) (Figure 5; Miyake et al., 2009). Compounds 1–3 at a concentration of 10 μM showed cytotoxic activity against A549 and MCF-7 cells with 42 ± 0.96% and 53 ± 1.03%, 32 ± 0.45% and 27 ± 0.53%, and 79 ± 0.89% and 48 ± 1.09% inhibition, respectively. Compound 3 was the most active against A549 cells with an IC_50_ value of 5.67 μM (IC_50_ = 0.61 μM for the positive control, adriamycin). Compounds **1**–**3** have been previously reported to show good antimicrobial and antimalarial bioactivity, inhibition of LPS (Lipopolysaccharide)-induced NO release and opioid and cannabinoid receptor binding activity, and cytotoxicity against SF-268, MCF-7, NCI-H460, HeLa, and HepG-2 cells (Hamasaki et al., 1981; Miyake et al., 2009; Gao et al., 2011, 2012; Kim et al., 2014; Dzoyem et al., 2015; Liang et al., 2018; Elissawy et al., 2019).

To the best of our knowledge, there have been no previous reports of compounds **1**–**3** outlining that they exhibit cytotoxicity against A549. In the present study, compound **3** demonstrated strong selective inhibition against A549. When the environmental pH changed, the fungi respond to the ambient pH and transmit pH signals to the nucleus through transcriptional regulators, thereby regulating a series of gene expressions including secondary metabolite gene clusters. It has also been reported that pH sensing in filamentous fungi relies mainly on the regulatory pathway PacC, a global regulator of fungi, which regulates the transcription of secondary metabolite-related genes, such as aflatoxin, cephalosporin, and penicillin (Brakhage, 2013). This is a complex regulation system and it was difficult to conclude if this regulation system was related to our experimental results. However, these results do suggest that acid stress can activate silent gene clusters to expand the diversity of secondary metabolites and this strategy will lead to potential medicinal discovery.

## Conclusion

Our investigation indicated that mangroves in Thailand are rich in aciduric and acidophilic microbial resources. These acid-tolerant microorganisms are potential medicinal resources. Acid stress can activate silent gene clusters to produce secondary metabolites that cannot be obtained under neutral conditions, which could be a useful strategy for expanding the diversity of available metabolites for potential medicinal use.

## Data Availability Statement

The datasets presented in this study can be found in online repositories. The names of the repository/repositories and accession number(s) can be found in Table 2.

## Author Contributions

All authors listed have made a substantial, direct and intellectual contribution to the work, and approved it for publication.

## Conflict of Interest

The authors declare that the research was conducted in the absence of any commercial or financial relationships that could be construed as a potential conflict of interest.
