# Supplementary material for: Bioactive Metabolites From Acid-Tolerant Fungi in a Thai Mangrove Sediment
Source: Front Microbiol. 2021 Jan 22;11:609952. doi: 10.3389/fmicb.2020.609952 (PMC7862741; doi:10.3389/fmicb.2020.609952)
Supplement: Supplementary file 1 [file Table_1.DOCX]

Supplementary Material

# Supplementary Figures and Tables

##
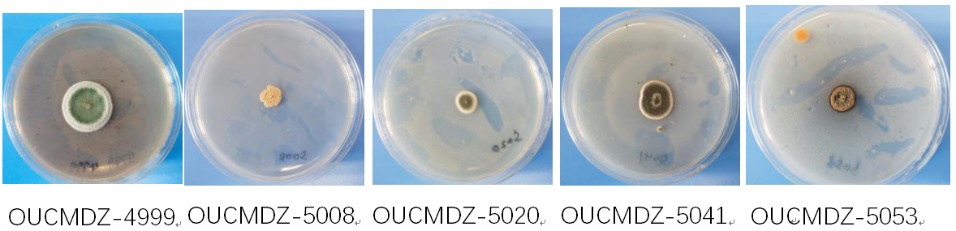

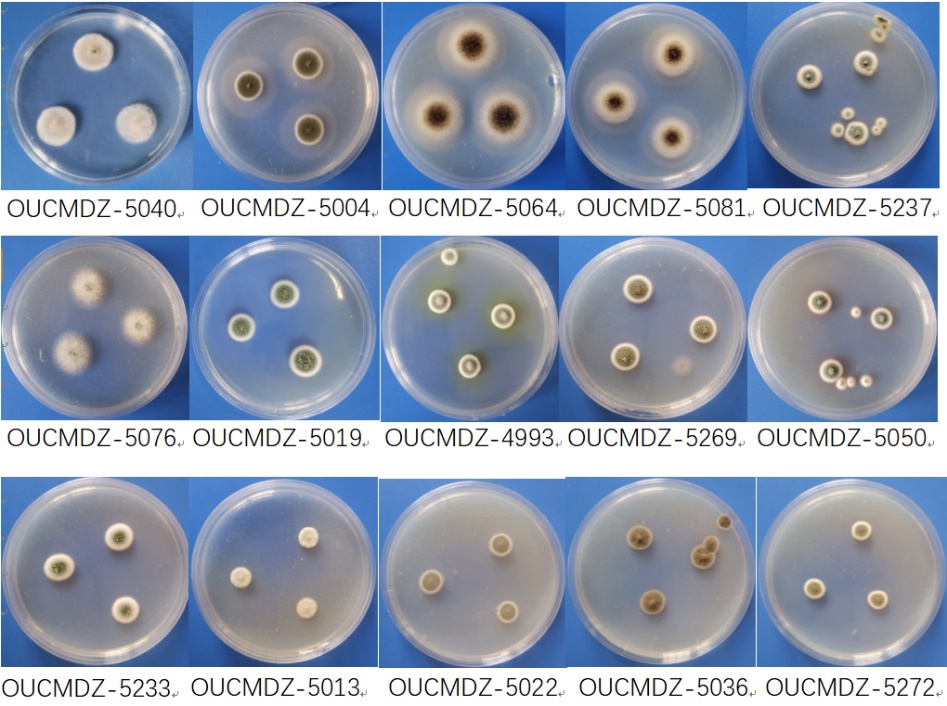

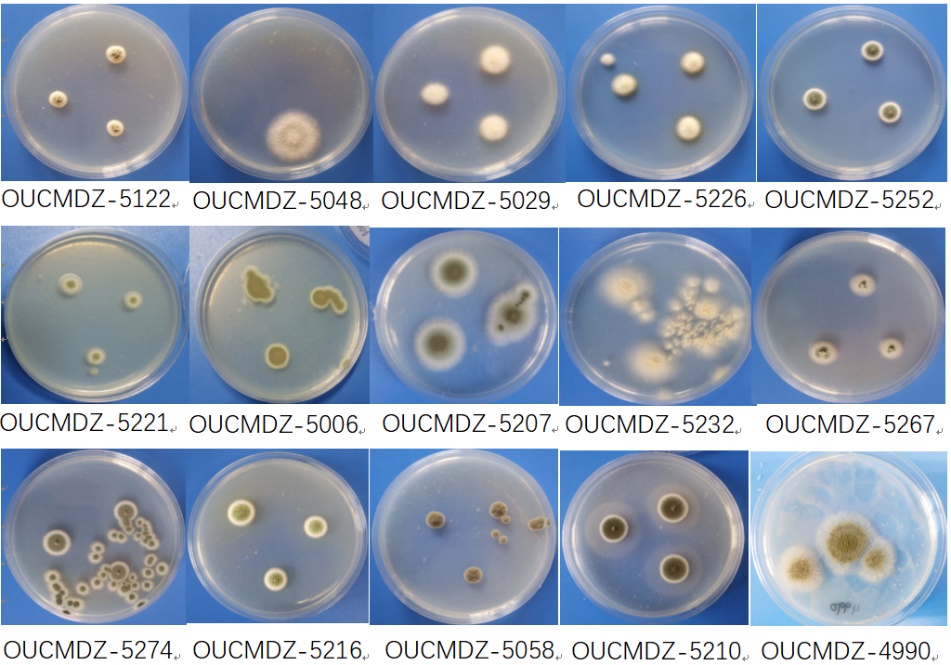
Supplementary Figures


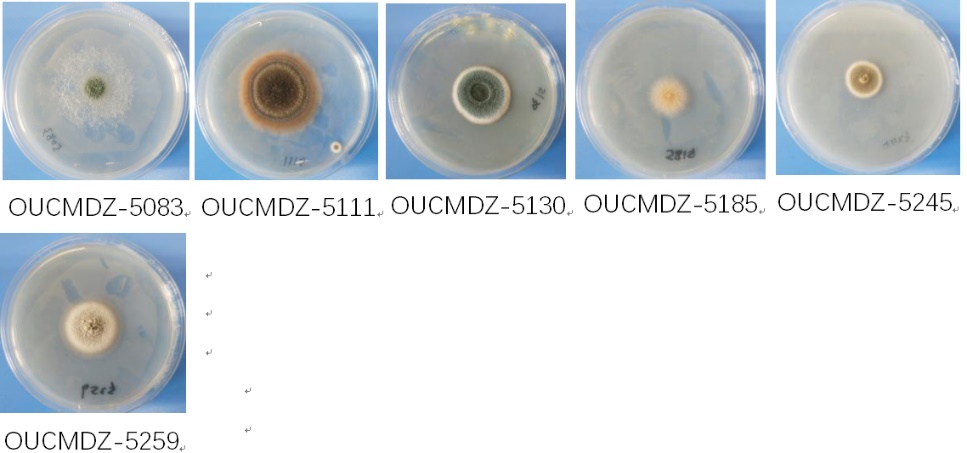


**Supplementary Figure S1.** The pictures of fungi


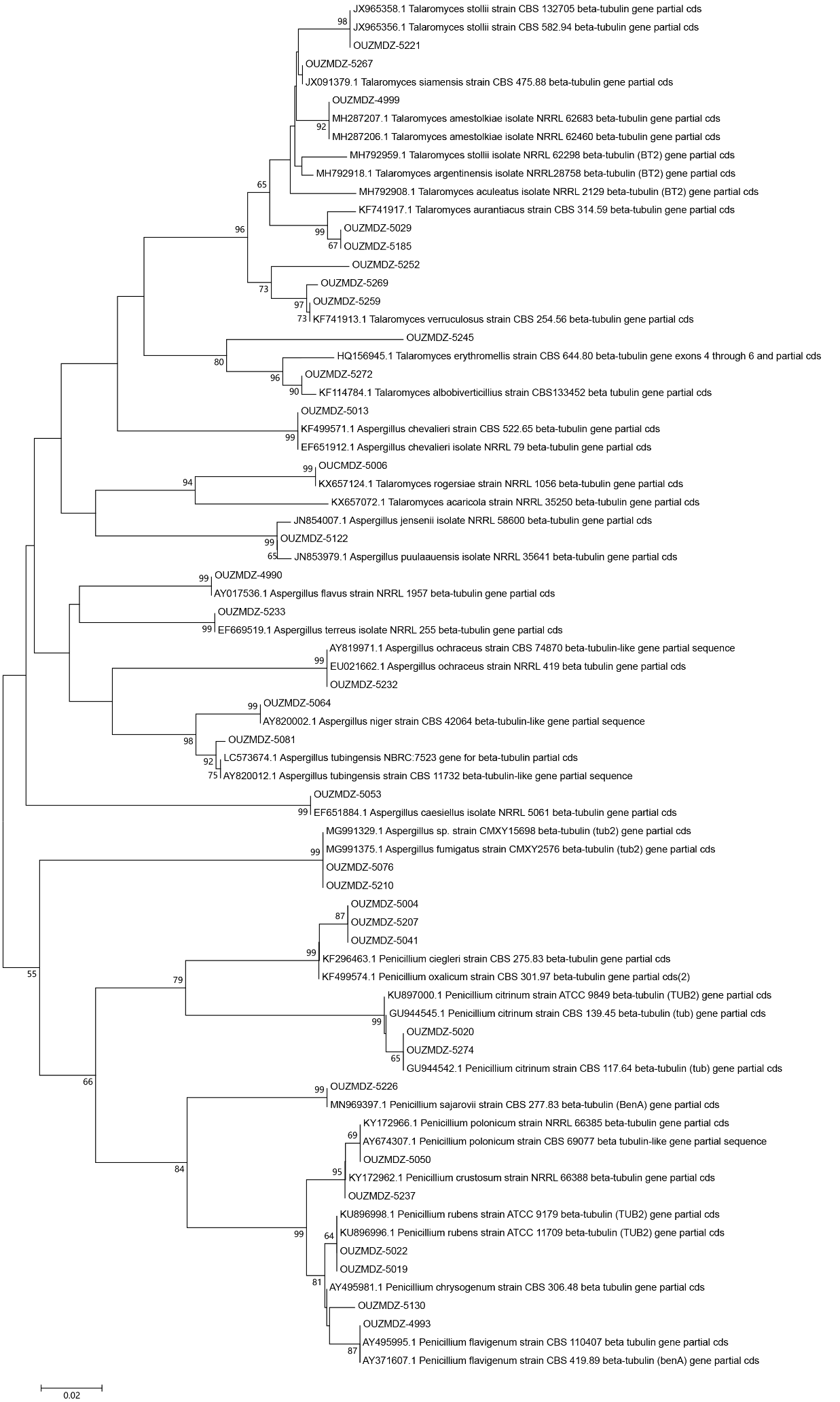


**Supplementary Figure S2.** Phylogenetic relationship of 33 representative fungal isolates based on beta tubulin gene sequences using the neighbor-joining method.


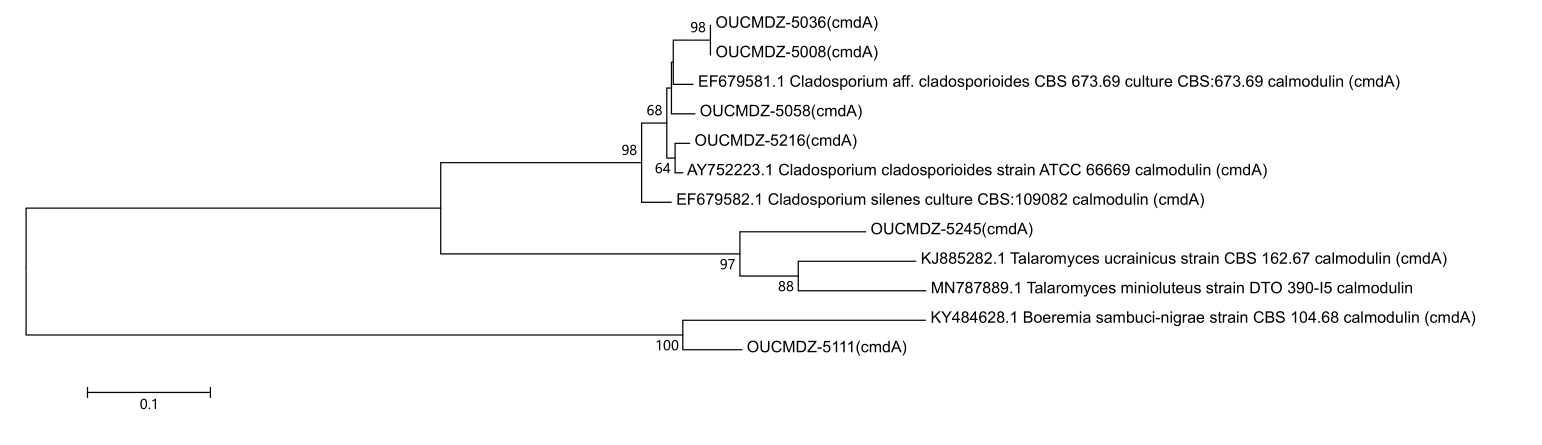


**Supplementary Figure S3.** Phylogenetic relationship of 6 representative fungal isolates based on calmodulin gene sequences using the neighbor-joining method.


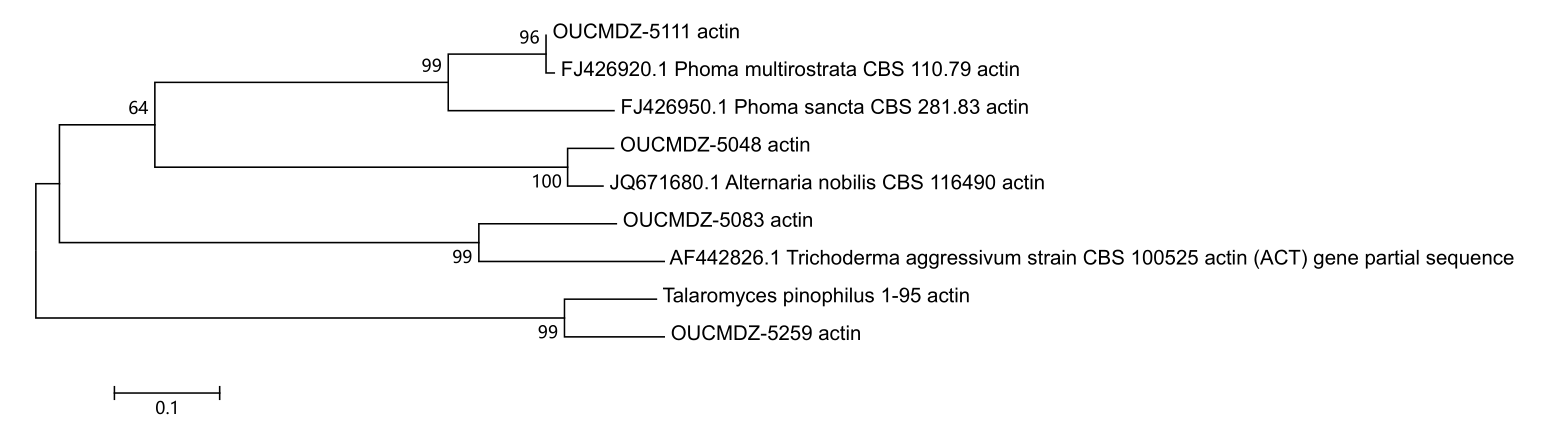


**Supplementary Figure S4.** Phylogenetic relationship of 4 representative fungal isolates based on actin gene sequences using the neighbor-joining method.

## Supplementary Tables

**Supplementary Table S1.** The fermentation conditions for the forty-one representative fungi

| PDA medium pH | Strain No. |
| --- | --- |
| pH3 | OUCMDZ-5020, OUCMDZ-5041, OUCMDZ-5050, OUCMDZ-5130, OUCMDZ-5019, OUCMDZ-5004, OUCMDZ-5022, OUCMDZ-5122, OUCMDZ-5013, OUCMDZ-5053, OUCMDZ-4990, OUCMDZ-5064, OUCMDZ-5081, OUCMDZ-5076, OUCMDZ-5029, OUCMDZ-5006, OUCMDZ-5185, OUCMDZ-4999, OUCMDZ-5058, OUCMDZ-5036, OUCMDZ-5008, OUCMDZ-5040, OUCMDZ-5048, OUCMDZ-5083 |
| pH7 | OUCMDZ-4993, OUCMDZ-5111 |
| pH3 and pH7 | OUCMDZ-5259, OUCMDZ-5274, OUCMDZ-5207, OUCMDZ-5210, OUCMDZ-5245, OUCMDZ-5233, OUCMDZ-5237, OUCMDZ-5216, OUCMDZ-5226, OUCMDZ-5232, OUCMDZ-5267, OUCMDZ-5269, OUCMDZ-5221, OUCMDZ-5272, OUCMDZ-5252 |

Fungi that grow better than pH7 at pH3 will be fermented in PDA medium at pH3; for fungi that grow better than pH3 at pH7, fermented in PDA medium at pH7; for pH3 and pH7 fungi with similar growth conditions were fermented in PDA medium at pH3 and pH7.

# Supplementary data

The sequences of β-tubulin, calmodulin, and actin gene.

> OUCMDZ-5019 beta-tubulin gene, partial cds

GGTAACCAAATCGGTGCTGCTTTCTGGTAAGTCTCGGAGCTTTTTTTTTCGCGTTGGGTATCAATTGACAAGTTGCTAACTGGATTACAGGCAAACCATCTCTGGCGAGCACGGTCTCGATGGCGATGGACAGTAAGTTTAACAGTGATAGGGATTCTGGTGGATCACACGTCTGATATCTTGCTAGGTACAATGGTACCTCCGACCTCCAGCTCGAGCGTATGAACGTCTACTTCAACCATGTGAGTACAATGACTGGGAATCGATTGATTGTGCATCATCTGATCGGACGTTTTTCTTTGACAATCTAGGCCAGCGGTGACAAGTACGTTCCCCGTGCCGTTCTGGTCGATTTGGAGCCCGGTACCATGGATGCTGTCCGCTCCGGTCCCTTCGGCAAGCTTTTCCGCCCCGACAACTTCGTCTTCGGTCAGTCCGGTGCTGGTAACAACTGGGCCAAGGGTCACTACACTGAGGGTA

> OUCMDZ-5020 beta-tubulin gene, partial cds

TGGTAACCAAAATCGGTGCTGCTTTCTGGTACGTGCTGCAAATCCTGAAAGATCAATTGTTGGATACAAAGAGCAATATACTGACCAATTCTATAGGCAAACCATTGCTGGCGAGCACGGCCTTGATGGCGATGGACAGTAAGTTCTTTCGACAAAGAAGTGTTTACGTTTTTTCGAGAATGGCGGTCTGATATTTTTGGGCAGCTACAACGGAACCTCCGATCTCCAGCTGGAGCGCATGAACGTCTACTTCACCCACGTAAGTGATACTGACCCCAATGCCATTGGAATCATTATCTAACCAACTATTGTTTGTTTTATCAATAGGCTTCAGGTGACAAGTATGTTCCCCGTGCCGTCCTGGTCGACTTGGAGCCCGGTACCATGGACGCTGTCCGTGCTGGTCCCTTCGGCAAGCTTTTCCGTCCCGACAACTTCGTTTTCGGTCAATCCGGTGCTGGTAACAACTGGGCCAAGGGTCACTAACCTGAGGGTA

> OUCMDZ-5210 beta-tubulin gene, partial cds

TGCTGCTTTCTGGTATGTCTTGACCTCAAAGCTTGGATGACGGGTGATTGGGATCTCTCATCTTAGCAGGCTACCTCCATGGGTTCAGCCTCACTGTCATGGGTATCAGCTAACAAATCTACAGGCAGACCATCTCTGGTGAGCATGGCCTTGACGGCTCTGGCCAGTAAGTTCGACCTATATCCTCCCAATTGAGAAAGCGGCAGAAACACGGAAAACAAGGAAGAAGCGGACGCGTGTCTGATGGGAAATAATAGCTACAATGGCTCCTCCGATCTCCAGCTGGAGCGTATGAACGTCTATTTCAACGAGGTGTGTGGATGAAACTCTTGATTTATACTATTTCGGCAACATCTCACGATCTGACTCGCTACTAGGCCAACGGTGACAAATATGTTCCTCGTGCCGTTCTGGTCGATCTCGAGCCTGGTACCATGGACGCTGTCCGTGCCGGTCCCTTCGGCGAGCTATTCCGTCCCGACAACTTCGTCTTCGGCCAGTCCGGTGCTGGTAACAACTGGGCCAAGGGTC

> OUCMDZ-5274 beta-tubulin gene, partial cds

TGGTAACCAAAATCGGTGCTGCTTTCTGGTACGTGCTGCAAATCCTGAAAGATCAATTGTTGGATACAAAGAGCAATATACTGACCAATTCTATAGGCAAACCATTGCTGGCGAGCACGGCCTTGATGGCGATGGACAGTAAGTTCTTTCGACAAAGAAGTGTTTACGTTTTTTCGAGAATGGCGGTCTGATATTTTTGGGCAGCTACAACGGAACCTCCGATCTCCAGCTGGAGCGCATGAACGTCTACTTCACCCACGTAAGTGATACTGACCCCAATGCCATTGGAATCATTATCTAACCAACTATTGTTTGTTTTATCAATAGGCTTCAGGTGACAAGTATGTTCCCCGTGCCGTCCTGGTCGACTTGGAGCCCGGTACCATGGACGCTGTCCGTGCTGGTCCCTTCGGCAAGCTTTTCCGTCCCGACAACTTCGTTTTCGGTCAATCCGGTGCTGGTAACAACTGGGCCAAGGGTCACTACACTGAGGGTA

> OUCMDZ-4990 beta-tubulin gene, partial cds

TACCCTCAGTGTAGTGACCCTTGGCCCAGTTGTTACCAGCACCGGACTGGCCGAAAACGA

AGTTGTCGGGACGGAAGAGCTGACCGAAGGGACCGGCACGGACGGCGTCCATGGTACCAG

GCTCAAGATCAACGAGGACGGCACGAGGGACATACTTGTTTCCGCTGGCCTGGAGAAGCG

GTCAGGAGTTGCAAAGCGTTTTCATAGATGCTGAAATTTTGAGGTACGCACCTCGTTGAA

GTAGACGTTCATACGCTCCAGCTGGAGATCGGAGGAGCCATTGTAACTATCCTTGCCGTC

AGATCCATTCCAACTTCTAATGCCATATGGTCGTCGTTCGTTCGAGGTGTATACAGGCTG

TACTTACACACCGGAGCCGTCAAGGCCGTGCTCGCCAGAGATGGTTTGCCTGCAAAGTTG

TTAGCAAACATGTGGCAGATATAACAGGACACATCCGAGATCATGCTTTTGAGGAGTTCC

TTGGTCCAAAGCATACTAACTCGAAGGCATTGAGACATACCAGAAAGCAGCACCGATTTG

GTTACCA

> OUCMDZ-4993 beta-tubulin gene, partial cds

TACCCTCAGGTGTAGTGACCCTTGGCCCAGTTGTTACCAGCACCGGACTGACCGAAGACG

AAGTTGTCGGGGCGGAAAAGCTTGCCGAAGGGACCGGAGCGGACAGCGTCCATGGTACCG

GGCTCCAAATCGACCAGAACGGCACGGGGAACGTACTTGTCACCGCTGGCCTAGATTGTC

AAAGAAAAACGTCCGATCAGATGATGCACGATTAATCGATTCCCGGTCATTATACTCACA

TGGTTGAAGTAGACGTTCATACGCTCGAGTTGAAGGTCGGAGGTACCATTGTACCTAGCA

AGATATCAGACCTGTGATCCACCAAAATCCCCATCACTGTTAAACTTACTGTCCATCGCC

ATCGAGACCGTGCTCGCCAGAGATGGTTTGCCTGTAATTCAGTTAGCAACTTGTCAATTG

ATACCCAACGCCGAGAAAAAAAAGCTCCGAGACTTACCAGAAAGCAGCACCGATTTGGTT

> OUCMDZ-4999 beta-tubulin gene, partial cds

TACCCTCAGGTGTAGTGACCCTTGGCCCAGTTGTTACCAGCACCGGACTGACCGAAAACA

AAGTTGTCGGGACGGAAGAGCTGACCAAAGGGACCAGCGCGGACGGCGTCCATGGTACCG

GGCTCCAAATCGACGAGGACGGCACGGGGGACGTATTTGTTGCCGGAGGCCTAGATGAAT

GAGTGTCTGTTCTATAGGAGTCGAGAGACTTTCTAACGCACCTCGTTGAAGTAGACGTTC

ATACGCTCCAACTGGAGGTCGGAGGAGCCATTGTAGCTGTCGATGATCAGATGTTGTCGG

ACTGTAGGTGTATTCGAACTGTGAAATACTTACACACCAGAGCCATCGAGACCGTGCTCA

GCAGAGATGATTTGCCTGGATAAGTCAGCGTGCTGTCGCGACAATTGATAGTAGCTTGGG

ACGAGTCAAACTCACCAGAAAGCAGCACCGATTTTGGTTACCA

> OUCMDZ-5004 beta-tubulin gene, partial cds

TACCCTCAGGTGTAGTGACCCTTGGCCCAGTTGTTACCAGCACCAGACTGGCCAAAGACG

AAGTTGTCGGGGCGGAAGAGCTTGCCAAAGGGACCGGCACGGACAGCGTCCATGGTACCA

GGCTCCAGGTCGACGAGAACGGCACGGGGAACATACTTGTCACCGCTAGCCTGGAAAATC

GAACGTTTCAATGATCAGGTTTGGGAGATTTCTTTGATTGGGAAGACGTTTTTCACGACT

TACGTGGGTGAAGTAGACGTTCATGCGCTCCAGCTGGAGGTCGGAGGAACCATTGTAGCT

AAACCGAAAAATATCAGACCGCCATTCTCTGTCGTCAGGATCCCATCGTGGGGGGGGGGC

AAGCTGTTGACTTACTGGCCATCGCCGTCAAGGCCGTGCTCACCAGCAATTGTTTGCCTG

AATATCGAGTCAGTTTGGTTCTCATATTAGATCGAATCACGACACAAGGAATTGTCGTGT

TGTGCTCGGGATATCCCAGGGAGGCGTGGCACGTACCAGAAAGCAGCACCGATTTGGTTA

CCA

> OUCMDZ-5013 beta-tubulin gene, partial cds

TACCCTCAGGTTAGTGACCCTTGGCCCAGTTGTTACCGGCACCGGACTGACCGAAAACGA

AGTTATCGGGACGGAAGAGCTGGCCGAAGGGACCGGCACGGACGGCGTCCATGGTACCGG

GCTCAAGGTCGACGAGGACGGCACGGGGGACATATTTGTTGTTGGAGGCCTGTCACTGTC

AGAACTGTTTCCCACACAGACACGAATGAATTAGGCAAACCTCGTTGAAGTAGACGTTCA

TACGCTCCAACTGGAGGTCGGAGGAGCCATTGTAGCTGCAATCCATTTAGATATCCATAT

CCGATACGCGTCCATCTCGGAGACCCGACTGTACTTACACACCAGAGCCGTCGAGACCGT

GCTCGCCGGAGATAGTCTGCCTGTGATACTGTTAGTATATCACCCAATCTGACTCTGATT

ATAATGCGGACATACCAGAAAGCAGCACCGATTTTGGTTACCA

> OUCMDZ-5022 beta-tubulin gene, partial cds

TACCCTCAGGGTGAGTGACCCTTAGCCCAGTTGTTACCAGCACCGGACTGACCGAAGACG

AAGTTGTCGGGGCGGAAAAGCTTGCCGAAGGGACCGGAGCGGACAGCATCCATGGTACCG

GGCTCCAAATCGACCAGAACGGCACGGGGAACGTACTTGTCACCGCTGGCCTAGATTGTC

AAAGAAAAACGTCCGATCAGATGATGCACAATCAATCGATTCCCAGTCATTGTACTCACA

TGGTTGAAGTAGACGTTCATACGCTCGAGCTGGAGGTCGGAGGTACCATTGTACCTAGCA

AGATATCAGACGTGTGATCCACCAGAATCCCTATCACTGTTAAACTTACTGTCCATCGCC

ATCGAGACCGTGCTCGCCAGAGATGGTTTGCCTGTAATCCAGTTAGCAACTTGTCAATTG

ATACCCAACGCGAAAAAAAAAGCTCCGAGACTTACCAGAAAGCAGCACCGATTTGGTTAC

CA

> OUCMDZ-5029 beta-tubulin gene, partial cds

TACCCTCAGGTGTAGTGACCCTTGGCCCAGTTGTTACCGGCACCGGATTGACCGAAAACA

AAGTTGTCGGGACGGAAGAGCTGACCAAAGGGACCAGCGCGGACAGCATCCATGGTACCG

GGCTCCAAGTCGACGAGGACGGCACGGGGAACGTACTTGTTGCCGGAGGCCTATATTCGA

GACATAAGTTCCAAGCATTCGATGGGTTTTCTGATGGTTGATTCCACACACCTCGTTGAA

GTAGACGTTCATACGCTCCAACTGGAGGTCGGAGGAGCCATTGTAGCTGTTGATGATCAG

ATGGTGTCGGATTATAACTGCATTCGAATCGTTTGCAACACTTACACACCGGAGCCATCG

AGACCGTGCTCAGCAGAGATGATTTGCCTGGAAAAGTCAGCTTGTTTGTGGCGACAATTG

ACCGAGTCTTTTTACACACCAGAAAGCAGCACCGATTTTGGTTACCA

> OUCMDZ-5053 beta-tubulin gene, partial cds

TACCCTCAGGGTTAGTGACCCTTGGCCCAGTTGTTACCAGCACCAGACTGGCCGAAGACG

AAGTTGTCGGGACGGAAAAGCTGACCGAACGGACCGGCACGGACGGCGTCCATGGTACCG

GGCTCAAGGTCAACCAGGACAGCACGGGGAACGTACTTGTCATTGCTGGCCTAATGAAAG

AATCAATATACGCATTCTCGAATTTTTCTCACCGGTAGGTGAATGAATGAGAACCATAAC

CACTAACCTCGTTGAAGTAGACGTTCATACGCTCCAGCTGCAGGTCGGAAGAGCCATTGT

AGCTGCGATTCGATTAGGAATTGACTCGTTTTGCGGGCTCGAATTTTGGAATTGGAACGA

TCGTGCTAGGCGAGAATGATAATACTTACTGACCAGAGCCGTCGAGACCGTGCTCGCCGG

AGATGGTTTGCCTGTAAATCAATTAGCATGCCACGCACACACCGCAATAGACCACCAAGT

CATCCCCCTAAGAGATCCAATTCGACTTTGCATAGGACTCTTTCTCAAAGGGAAATCTCA

TGGATAGCAGGGAACATACCAGAAAGCAGCACCGATTTGGTTACCA

> OUCMDZ-5064 beta-tubulin gene, partial cds

CCTCAGGGTCGGTGACCCTTGGCCCAGTTGTTACCAGCACCGGACTGGCCGAAGACGAAG

TTGTCGGGGCGGAAGAGCTGGCCGAAAGGACCGGCACGGACGGCGTCCATGGTACCGGGC

TCGAGGTCGACGAGGACGGCACGAGGAACATACTTGTTACCGCTAGCCTGCTGAAGTGAT

TAGGACATTGATGATATTGTCGTGTAAAAGCAAAGGTCCGATGGATCTCACCTCGTTGAA

GTAGACGTTCATGCGCTCCAGCTGGAGGTCGGAGGTGCCATTGTAACTATCCTGCGTGTC

AGAAGACCCAACCCTTTGCCCATTGTTGACCAATTGAGAGGTGTGAAAAAGTTGCACTTA

CACACCGGAGCCGTCAAGGCCGTGCTCGCCAGAGATGGTCTGCCTGTTGAATTGTTAGAC

GTGTTCTTAGACCGATAGCATGTACCTAATGGACAACATCTGAACTCAAGCTGAGATAGC

CTAAGAGACGATGTTCCATCCCCAATCCAATGGCAGTTGTATACGTACCAGAAAGCAGCA

> OUCMDZ-5076 beta-tubulin gene, partial cds

TACCCTCAGGTGTAGTGACCCTTGGCCCAGTTGTTACCAGCACCGGACTGGCCGAAGACG

AAGTTGTCGGGACGGAATAGCTCGCCGAAGGGACCGGCACGGACAGCGTCCATGGTACCA

GGCTCGAGATCGACCAGAACGGCACGAGGAACATATTTGTCACCGTTGGCCTAGTAGCGA

GTCAGATCGTGAGATGTTGCCGAAATAGTATAAATCAAGAGTTTCATCCACACACCTCGT

TGAAATAGACGTTCATACGCTCCAGCTGGAGATCGGAGGAGCCATTGTAGCTATTATTTC

CCATCAGACACGCGTCCGCTTCTTCCTTGTTTTCCGTGTTTCCGCCGCTTTCTCAATTGG

GAGGATATAGGTCGAACTTACTGGCCAGAGCCGTCAAGGCCATGCTCACCAGAGATGGTC

TGCCTGTAGATTTGTTAGCTGATACCCATGACAGTGAGGCTGAACCCATGGAGGTAGCCT

GCTAAGATGAGAGATCCCAATCACCCGTCATCCAAGCTTTGAGGTCAAGACATACCAGAA

AGCAGCACCGATTTTGGTTACCA

> OUCMDZ-5081 beta-tubulin gene, partial cds

TACCCTCAGGTGTAGTGACCCTTGGCCCAGTTGTTACCAGCACCGGACTGGCCGAAGACG

AAGTTGTCGGGGCGGAAGAGCTGGCCGAAGGGACCGGCACGGACGGCGTCCATGGTACCG

GGCTCGAGATCGACGAGGACGGCACGGGGGACATACTTGTTACCGCTAGCCTGCTGAAGT

GGTCAGGACATTGATGATATTGTCGTGAAAAAACTCAGGGACGGTGTGATATAACCTCGT

TGAAGTAGACGTTCATGCGCTCCAGCTGGAGGTCGGAGGTGCCATTGTAACTATCCTGCG

CGTCAGGAAACCCAATCCTTTCCACACTGTTGACCAATTGAGAGGTGTGAAAAAGTTGTA

CTTACACACCGGAGCCGTCAAGGCCGTGCTCGCCAGAGATGGTCTGCCTGTTGAGTTGTT

AGACGTGTTCTTAACCCGATAGCTATATACCTGATGGATAACATCTGAACTCAAGCTAAG

ACAGCTTGAGAGATGATGTTCCATCCCCAATCCAGTGGCAGTGAATACGTACCAGAAAGC

AGCACCGATTTGGTTACCA

> OUCMDZ-5122 beta-tubulin gene, partial cds

TACCCTCAGTGTAGTGACCCTTGGCCCAGTTGTTACCAGCACCGGACTGGCCAAAGACGA

AGTTGTCGGGACGGAAGAGCTGACCGAAGGGACCGGCACGGACAGCGTCCATGGTACCGG

GCTCGAGATCGACGAGGACGGCACGAGGAACGTACTTGTTGCCGCTGGCCTCGTTGAAGT

AGACGTTCATACGCTCGAGCTGGAGGTCGGAGGTACCATTGTAACTGCATTATATCAGGG

GATGTGTTCTGTCTCGTGTTTTGATCGAGTCCTGGACGGGTTGTACTCACACACCGGAGC

CATCGAGGCCGTGCTCACCGGAGATGGTCTGCCTATAGAGTCGTTAGCAAAAAGCACGAA

AGGAAATACCATCTGAAATGGATGAAATTTTCGACGCACCAGAAAGCAGCACCGATTTGG

TTACCA

> OUCMDZ-5130 beta-tubulin gene, partial cds

AACCAAATCGGTGCTGCTTTCTGGTAAGTCTCGGAGCTTTTTTTTCGCGTTGGGTATCAATTGACAAGTTGCTAACTGGATTACAGGCAAACCATCTCTGGCGAGCACGGTCTCGATGGCGATGGACAGTAAGTTTAACAGTGATGGGGATTTTGGTGGATCACACGTCTGATATCTTGCTAGGTACAATGGTACCTCCGACCTCCAGCTCGAACGCATGAACGTCTACTTCAACCATGTGAGTACAATGGCTGGGAATCGATTAATTGTGCATCATCTGATCGGGCGTTTTTCTTTGACAATCTAGGCCAGCGGTGACAAGTACGTTCCCCGTGCCGTTCTGGTCGATTTGGAGCCCGGTACCATGGACGCTGTCCGCTCCGGTCCCTTCGGCAAGCTTTTCCGCCCCGACAACTTCGTCTTCGGTCAGTCCGGTGCTGGTAACAACTGGGCCAAGGGTCACTACACTGAGGGTA

> OUCMDZ-5185 beta-tubulin gene, partial cds

TACCCTCAGGTGTAGTGACCCTTGGCCCAGTTGTTACCGGCACCGGATTGACCGAAAACA

AAGTTGTCGGGACGGAAGAGCTGACCAAAGGGACCAGCGCGGACAGCATCCATGGTACCG

GGCTCCAAGTCGACGAGGACGGCACGGGGAACGTACTTGTTGCCGGAGGCCTATATTCGA

GACATAAGTTCCAAGCATTCGATGGGTTTTCTGATGGTTGATTCCACACACCTCGTTGAA

GTAGACGTTCATACGCTCCAACTGGAGGTCGGAGGAGCCATTGTAGCTGTTGATGATCAG

ATGGTGTCGGATTATAACTGCATTCGAATCGTTTGCAACACTTACACACCGGAGCCATCG

AGACCGTGCTCAGCAGAGATGATTTGCCTGGAAAAGTCAGCTTGTTTGTGGCGACAATTG

ACCGAGTCTTTTTACACACCAGAAAGCAGCACCGATTTTGGTTACCA

> OUCMDZ-5221 beta-tubulin gene, partial cds

CCTCAGGTTAGTGACCCTTGGCCCAGTTGTTACCAGCACCGGACTGACCGAAGACAAAGT

TGTCGGGACGGAAGAGCTGACCAAAGGGACCAGCACGGACGGCGTCCATGGTACCGGGCT

CCAAGTCGACGAGGACAGCACGGGGGACGTATTTGTTGCCGGAGGCCTAGCTGAATAAGT

GTTTGTTCTGCAGGAGTCGAGAAACTTTCAAACGCACCTCGTTGAAGTAGACGTTCATAC

GCTCCAACTGGAGGTCGGAGGAGCCATTGTAGCTGTCGATGATCAGATAACCTCGGACAG

TAGGTGTATTCGAATTGTGAAATACTTACACACCAGAGCCATCGAGACCGTGCTCAGCAG

AGATAATTTGCCTGTACAAGTCAGCGTGCCGCCGCGACAATTGATAGAAAGTTTGGGTCG

AGTCAAACTCACCAGAAAGCAGCACCGATTTTGGTTACCA

> OUCMDZ-5226 beta-tubulin gene, partial cds

TACCCTCAGTTTAGTGACCCTTGGCCCAGTTGTTACCAGCACCGGACTGACCGAAGACGA

AGTTGTCGGGACGGAAGAGCTTGCCGAAGGGACCGGAGCGGACGGCATCCATGGTACCGG

GCTCCAAATCGACGAGGACAGCACGGGGGACGTATTTGTCACCGCTAGCCTGAGCGGTCA

AAGAAAGAAGGTTAGACAACGCACGATTTAAAAATCAAAGCAATTCTACTCACATGGTTG

AAGTAGACGTTCATACGCTCCAACTGGAGGTCGGAGGTACCGTTGTACCTAGGAAGATAT

CAGAGGTCTGATTCAACTGGAATCTTATCTCATTGAACTTACTGTCCATCGCCATCGAGA

CCGTGCTCACCGGAGATGGTTTGCCTGTAAACCAGTTAGCAATTTGTCAATTGATACCCA

ACACCGAAAAAAAAAGTTGAGGATGTTGCACGTACCAGAAAGCAGCACCGATTTTGGTTA

CCA

> OUCMDZ-5232 beta-tubulin gene, partial cds

TACCCTCAGGGTGAGTGACCCTTGGCCCAGTTGTTACCGGCACCAGACTGGCCGAAGACG

AAGTTGTCGGGGCGGAAAAGCTGACCGAAGGGACCGGCACGGACAGCGTCCATGGTACCG

GGCTCAAGATCGACCAGAACGGCACGGGGAACATACTTGCCACCGGAAGCCTAGAAGATG

AAGAAAAAAGCGTGGGTTAGAGCTTTGTACGGGGCGTTTCGAATAGGCGAAAGGAGATAG

AAAATTTTCGGGCAACGAACCTCGTTGAAGTAGACGTTCATGCGCTCCAGCTGAAGGTCG

GAGGAGCCATTGTAACTGTTCATCCCATCAGACAATCAATCCCACCATGGTCGTTTCTTT

CTTTTCCTCTATTTTCGACTCTGATTTCGATGGGTGTAAACGCGGGATGTACTTACACAC

CGGCGCCGTCAAGGCCGTGCTCGCCAGAGATGTTTTGCCTATTCACCTATCAGTAAACAA

GTATCAAGGATTGAGAGTCATAAGTCCTAAGGTATCGAAGATGATCCTGGATAGCCCAGA

CTGCCAATGCCAACGTGTAGACTTACCAGAAAGCAGCA

> OUCMDZ-5237 beta-tubulin gene, partial cds

TACCCTCAGTGTAGTGACCCTTGGCCCAGTTGTTACCAGCACCGGACTGACCGAAGACGA

AGTTGTCGGGGCGGAAAAGCTTGCCGAAGGGACCGGAGCGGACAGCGTCCATGGTACCAG

GCTCCAAATCGACGAGAACGGCACGGGGAACGTACTTGTCACCGCTGGCCTAGATTATCA

AGGAAAACATCCGATCAGATGATGCACTATTATTCGGTTTCCTGTCGTTGGACTCACATG

GTTGAAGTAGACGTTCATACGCTCGAGCTGGAGGTCGGAGGTACCATTGTACCTAGGAAG

ATATCAGATGTGTAATCCACCGGAAACCCCTATCACTGTTAAAACTTACTGTCCATCGCC

ATCGAGACCGTGCTCGCCAGAGATGGTTTGCCTGTAATCCAGTTAGGAACCTGTCAATTG

ATACCCAACGCGAAAAAAAAAAGCTCGGCACTTACCAGAAAGCAGCACCGATTTGGTTAC

CA

> OUCMDZ-5245 beta-tubulin gene, partial cds

TACCCTCAGGTTAGTGACCCTTGGCCCAGTTGTTACCGGCACCAGACTGGCCGAAAACAA

AGTTGTCGGGACGGAAGAGCTGTCCAAAGGGACCGGCACGGACGGCATCCATGGTACCGG

GCTCCAAGTCGACGAGGACGGCACGAGGGACATACTTGTTACCGCTGCCCTGTATTCGGT

TGTGAGTACTGTTGTTCTGCTTGTTTCGTTTGATATCGAATCCTGACGCACCTCGTTGAA

GTAGACGTTCATACGCTCCAACTGGAGGTCGGAGGATCCATTGTAACTGTTTTTTTTCTG

CTGTCAGACGTGTCGTTCTTTCGTGATCGATCGAAAACGCATGGAAACACTCACACGCCA

GAGCCGTCGAGACCGTGTTCGCCGGAGATGATTTGCCTAGAAGAAGTCAGCGTGGTTTGC

CTCTTGACGCCAGAATGCAATGTGCAGTGCTCACCAGAAAGCAGCACCGATTTTGGTTAC

CA

> OUCMDZ-5252 beta-tubulin gene, partial cds

TACCCTCAGTTTAGTGACCCTTGGCCCAGTTGTTACCAGCACCGGACTGACCGAAAACAA

AGTTGTCGGGACGGAAGAGCTGGCCAAAGGGACCAGCGCGGACGGCGTCCATGGTACCGG

GCTCCAAGTCGACGAGGACAGCACGAGGGACATACTTGTTGCCGGAAGCCTATGCAATTG

ATGAGTCTGTGGTCTGTGTTTCGTTTGAGTCGAGTGATTCTAACGCACCTCGTTGAAGTA

AACGTTCATACGCTCCAACTGGAGGTCGGAGGAGCCATTGTAGCTGTTGATTATCAGATA

GTGTCGAATTGTAGGATTTCGATTCGTATCAATACTTACACACCGGAGCCATCGAGACCG

TGCTCAGCGGAGATGATTTGCCTGGAAAGAGTCAGCGTGTTGTCGCGAGAATTGACCGAA

AGTTTGGGTCGAGAGTCAACACTCACCAGAAAGCAGCACCGATTTTGGTTACCA

> OUCMDZ-5267 beta-tubulin gene, partial cds

TACCCTCAGTGTAGTGACCCTTGGCCCAGTTGTTACCAGCACCGGACTGACCGAAAACAA

AGTTGTCGGGACGGAAGAGCTGACCAAAGGGACCAGCGCGGACGGCGTCCATGGTACCGG

GCTCCAAGTCGACGAGGACAGCACGGGGGACGTATTTGTTGCCGGAGGCCTAATACCATT

GTAAGTTTGTTTCGTCATATGGTGGATTGGTACGACGCACCTCGTTGAAGTAGACGTTCA

TACGCTCCAACTGGAGGTCGGAGGAGCCATTGTAGCTGTTGACGATCAGATATTGTCGGA

TTGTAGCTGGATTCGAGGTCGTGAATACTTACACACCAGAGCCATCGAGACCGTGCTCAG

CAGAGATGATTTGCCTGAAAAAGTCAGCGTGTTGTCGCGACAATTGATAGAAAGTTTGGG

TCGAGTCAAACTCACCAGAAAGCAGCACCGATTTGGTTACCA

> OUCMDZ-5272 beta-tubulin gene, partial cds

TACCCTCAGGTTGAGTGACCCTTGGCCCAGTTGTTACCGGCACCAGACTGGCCGAAAACA

AAGTTGTCGGGACGGAAGAGCTGGCCAAAGGGACCAGCGCGGACGGCGTCCATGGTACCG

GGTTCCAAGTCGACGAGGACGGCACGGGGGACATACTTGTTTCCGCTGGCCTGTATTGAA

TTCGATTGTGAGTACTTTTTTCATGGTCCTTCGTTTTGGTATCAAGTTCGACGCACCTCG

TTGAAGTAAACGTTCATACGCTCCAACTGGAGGTCGGAGGAGCCATTGTAACTGGATTTT

GTCAGACGGTTGCTTTTCGATTAATCGAGAATCGTATCGAAATACTCACACTCCGGAGCC

GTCGAGACCGTGCTCGCCAGAGATGATTTGCCTAGAAACAGTGAGCACGTTGATTTTTCA

AGCTGACTGATATTTCATGATACTCACCAGAAAGCAGCAC

> OUCMDZ-5269 beta-tubulin gene, partial cds

TTACCCTCAGGTTAGTGACCCTTGGCCCAGTTGTTACCAGCACCGGACTGACCGAAAACA

AAGTTGTCGGGACGGAAGAGCTGACCGAAGGGACCAGCGCGGACGGCGTCCATGGTACCG

GGCTCCAAGTCGACGAGGACAGCACGGGGGACATATTTGTTGCCGGAAGCCTATCGAGGG

ATGAGTTTTGTTCGATTTATCTGGTGGATTGGCTGACGCACCTCGTTGAAGTAAACGTTC

ATACGCTCCAACTGGAGGTCGGAGGAGCCATTGTAGCTGTTGATCATCAGATATCGTCGG

ACTGCAGCTGGATTCGAATCGTGTTATACTTACACACCGGAGCCGTCGAGACCGTGCTCA

GCAGAGATAATTTGCCTGAAAGAGTCAGTGTGCTGTCGCAACAATTGATCGAAAGTTGGG

TTCCAGAGTCAAACTCACCAGAAAGCAGCACCGATTTTGGTT

> OUCMDZ-5050 beta-tubulin gene, partial cds

TGGTAACCAAATCGGTGCTGCTTTCTGGTAAGTGCCGAGCTTTTTTTTCTTCTTCGCGTTGGGTATCAATTGACAGGTTACTAACTCGATTACAGGCAAACCATCTCTGGCGAGCACGGTCTCGATGGCGATGGACAGTAAGTTTTAATGGTGATGTGGGTTTCCGGTAGATCACACGTCTGATATCTTGCTAGGTACAATGGTACCTCCGACCTCCAGCTCGAGCGTATGAACGTCTACTTCAACCATGTGAGTCCAATCACTGGAAACCGAATAATCGTGCATCATCTGATCAGATGTTTTTCTTTGATATCTAGGCCAGCGGTGACAAGTACGTTCCCCGTGCCGTTCTCGTCGATTTGGAGCCTGGTACCATGGACGCTGTCCGCTCCGGTCCTTTCGGCAAGCTTTTCCGCCCCGACAACTTCGTCTTCGGTCAGTCCGGTGCTGGTAACAACTGGGCCAAGGGTCACTAACCTGAGGGTA

> OUCMDZ-5233 beta-tubulin gene, partial cds

TGGTAACCAAAATCGGTGCTGCTTTCTGGTACGTCTGGAATCAACCTGGGGAATGCTGGCTCTCGTGGGATGCAGAGTCTTACGGACATGCGTCCTCGGGCTAAAAAGGGTTCCGTGGTGGCATGATGCTGACAACTGTACAGGCAAACCATCTCTGGCGAGCACGGCCTTGATGGCTCCGGTGTGTAAGTGTCACCGACGCCCGCTCAATGGGCTCCCATAATGGAGGTTTACACGACGATGGACGATTCTGATTTGGAAACAGCTTCAATGGCTCCTCCGACCTCCAGCTCGAGCGCATGAACGTCTACTTCAACGAGGTATGTCCCTTCCACACCATCCTGGGACAGATTCTCCACGCTCCAAAGACCTCGACACTAATTCCGATCCCCTTTAGGCCAGCGGAAACAAGTATGTTCCTCGTGCCGTCCTCGTTGACCTTGAGCCCGGTACCATGGACGCCGTCCGTGCCGGTCCCTTCGGTCAGCTCTTCCGTCCCGACAACTTCGTCTTCGGCCAGTCTGGTGCCGGTAACAACTGGGCCAAGGGTCACTACACCTGAGGGTA

> OUCMDZ-5259 beta-tubulin gene, partial cds

TGGTAACCAAAATCGGTGCTGCTTTCTGGTGAGTTTGACTCTGGAACCCAACTTTCGATCAATTGTTGCGACAGCACACTGACTCTTTCAGGCAAATCATCTCTGCTGAGCACGGTCTCGACGGCTCCGGTGTGTAAGTATAACACGATTCGAATCCAGCTGCAGTCCGACGATATCTGATGATCAACAGCTACAATGGCTCCTCCGACCTCCAGTTGGAGCGTATGAACGTTTACTTCAACGAGGTGCGTCAGACCAATCCACCAGATAAATCGAACAAGACTCATCCCTCGATAGGCTTCCGGCAACAAATATGTCCCCCGTGCTGTCCTCGTCGACTTGGAGCCCGGTACCATGGACGCCGTCCGCGCTGGTCCCTTCGGTCAGCTCTTCCGTCCCGACAACTTTGTTTTCGGTCAGTCCGGTGCTGGTAACAACTGGGCCAAGGGTCACTCACCTGAGGGTA

> OUCMDZ-5207 beta-tubulin gene, partial cds

TGGTAACCAAATCGGTGCTGCTTTCTGGTACGTGCCACGCCTCCCTGGGATATCCCGAGCACAACACGACAATTCCTTGTGTCGTGATTCGATCTAATATGAGAACCAAACTGACTCGATATTCAGGCAAACAATTGCTGGTGAGCACGGCCTTGACGGCGATGGCCAGTAAGTCAACAGCTTGCCCCCCCCCCACGACGGGATCCTGACGACAGAGAATGGCGGTCTGATATTTTTCGGTTTAGCTACAATGGTTCCTCCGACCTCCAGCTGGAGCGCATGAACGTCTACTTCACCCACGTAAGTCGTGAAAAACGTCTTCCCAATCAAAGAAATCTCCCAAACCTGATCATTGAAACGTTCGATTTTCCAGGCTAGCGGTGACAAGTATGTTCCCCGTGCCGTTCTCGTCGACCTGGAGCCTGGTACCATGGACGCTGTCCGTGCCGGTCCCTTTGGCAAGCTCTTCCGCCCCGACAACTTCGTCTTTGGCCAGTCTGGTGCTGGTAACAACTGGGCCAAGGGT

>OUCMDZ-5006 beta-tubulin gene, partial cds

TGGTGAGTTGCGGGATAAACAACGGTACAAAAAAAAAAAAAAGAAACCACTCGCTGACGTCGTACAGGCAAACCATCTCTGGCGAGCACGGCCTCGATGGTTCCGGAGTGTGAGTGATGCGTGATTGTCACATTTTCGAGAACGAACCCTGATCGTATCCAGTTACAATGGCTCCTCCGACCTCCAGTTGGAGCGTATGAACGTCTACTTCAACGAGGTGCGTGTTATCTTCCCCACGACATTTGGGAATATACTCATATCGTATAGGCTAGCGGCAACAAGTATGTCCCCCGTGCCGTTCTCGTCGATTTGGAGCCTGGCACCATGGACGCTGTCCGCTCCGGTCCCTTCGGTCAGCTCTTCCGTCCCGACAACTTTGTGTTCGGC

>OUCMDZ-5041 beta-tubulin gene, partial cds

TGGTAACCAAATCGGTGCTGCTTTCTGGTACGTGCCACGCCTCCCTGGGATATCCCGAGCACAACACGACAATTCCTTGTGTCGTGATTCGATCTAATATGAGAACCAAACTGACTCGATATTCAGGCAAACAATTGCTGGTGAGCACGGCCTTGACGGCGATGGCCAGTAAGTCAACAGCTTGCCCCCCCCCCACGATGGGATCCTGACGACAGAGAATGGCGGTCTGATATTTTTCGGTTTAGCTACAATGGTTCCTCCGACCTCCAGCTGGAGCGCATGAACGTCTACTTCACCCACGTAAGTCGTGAAAAACGTCTTCCCAATCAAAGAAATCTCCCAAACCTGATCATTGAAACGTTCGATTTTCCAGGCTAGCGGTGACAAGTATGTTCCCCGTGCCGTTCTCGTCGACCTGGAGCCTGGTACCATGGACGCTGTCCGTGCCGGTCCCTTTGGCAAGCTCTTCCGCCCCGACAACTTCGTCTTTGGCCAGTCTGGTGCTGGTAACAACTGGGCCAAGGGTCACTACACCTGAGGGTA

>5036 calmodulin (cmdA) gene, partial cds

TGAGTTCAAGGAGGCCTTCTCCCTCTTCGTAAGTGACTGCCTAACCCCCGTATTTGTGGTGGGAAGCGCGTGAGGCTGACTCTTCGGAACAGGACAAGGACGGCGATGGTACGAATCTCGCAGCAACTACCTACAATACCGCCTACTAACACACATCTGACAGGACAAATCACCACCAAGGAGCTCGGCACCGTGATGCGCTCGCTCGGCCAGAACCCCTCTGAGTCTGAGCTGCAGGACATGATCAACGAGGTCGACGCCGACAACAACGGCACCATTGACTTCCCCGAGTTCCTCACCATGATGGCCAGAAGATGA

>5058 calmodulin (cmdA) gene, partial cds

TGAGTTCAAGGAGGCCTTCTCCCTCTTTGTAAGTGACTGCCTAAACCCCGTATTTGTGGTGGGAAGCGCGTGAGGCTGACTCTTCGAAACAGGACAAGGACGGCGATGGTACGAAACATGAAGCGACCACCTACAATACCGCCTACTAATACACCTCTGACAGGACAAATCACCACCAAGGAGCTCGGCACCGTGATGCGCTCGCTCGGCCAGAACCCCTCCGAGTCTGAGCTGCAGGACATGATCAACGAGGTTGACGCCGACAACAACGGCACCATTGACTTCCCCGAGTTCCTCACCATGATGGCCAGAAGATGA

>5111 calmodulin (cmdA) gene, partial cds

TGAGTTCAAGGAGGCCTTCTCCCTCTTCGTAAGTATTTCTCCAGCCCCTGTGCCGCGTGTTGTTGCCTGGCCGCCTGGGGGCATTTGCTCCTGTCAACAATGGCTTGCTGACCGCCTCGTGAACAGGACAAGGATGGCGATGGTTGGTAGCCCCTCCCCAGCACGAGCGCGCGACGGAAGACGATTCATCGAGACGCCCCTCGATCACATGCATGGCAAATGCCGCAGATGTTCTGTTGCTAGCCTCGAGTGCCCCTGTACTGCGGCACGCGTCATCAGCCACAACACATCTTCAAGAGCGCATAGCTGACAATGGTGTCTTACAGGCCAGATCACCACCAAGGAGCTCGGCACTGTCATGCGCTCGCTTGGCCAGAACCCCAGCGAGTCTGAGCTTCAGGACATGATCAACGAGGTTGACGCCGACAACAACGGCACCATTGACTTCCCCGGTACGGACCCAGCACTTCCAATGAAGGAGCCTTGGCTGACGGCTTTTCTCTAGAGTTCCTTACCATGATGGCCAGAAGATG

>5126 calmodulin (cmdA) gene, partial cds

TGAGTTCAAGGAGGCCTTCTCCCTCTTCGTAAGTGACTGCCTAACCCCCGTGTTCGTGCTGAGAAGCGCGCGAGGCTGACTCTTCGAAACAGGACAAGGACGGCGATGGTACGAAACGTGCCGCAAACACCTACAATACCGCCTACTAATACACCTCTGACAGGACAAATCACCACCAAGGAGCTCGGCACCGTGATGCGCTCGCTCGGCCAGAACCCCTCCGAGTCTGAGCTGCAGGACATGATCAACGAGGTCGACGCCGACAACAACGGCACCATTGACTTCCCCGAGTTCCTCACCATGATGGCCAGAAGATGA

>5008 calmodulin (cmdA) gene, partial cds

GAGTTCAAGGAGGCCTTCTCCCTCTTCGTAAGTGACTGCCTAACCCCCGTATTTGTGGTGGGAAGCGCGTGAGGCTGACTCTTCGGAACAGGACAAGGACGGCGATGGTACGAATCTCGCAGCAACTACCTACAATACCGCCTACTAACACACATCTGACAGGACAAATCACCACCAAGGAGCTCGGCACCGTGATGCGCTCGCTCGGCCAGAACCCCTCTGAGTCTGAGCTGCAGGACATGATCAACGAGGTCGACGCCGACAACAACGGCACCATTGACTTCCCCGAGTTCCTCACCATGATGGCCAGAAGATGAT

>5245 calmodulin (cmdA) gene, partial cds

CGATAGAGGGCATAACGTGGCGCAGCTCAGCTGCAGAAATGAATCCGTTGTTGTCGCGGT

CGAAAACCTTGAACGCCTCACGGATTTCTTCCTCGGAATCGGTGTCCTTCATTTTGCGGG

CCATCATTGTCAAGAATTCTACGCCAGTTAGTCGGACAGCATCTCAAAGAAGCCTCTTCA

TGCAGGCTGCCGTACCAGGAAAGTCAATTGTGCCATTGTTGTCAGCATCAACTTCGTTGA

TCATGTCCTGCAATTCTGACTCCGAGGGGTTCTGGCCGAGCGATCGCATGACAGTGCCAA

GCTCTTTTGTCGTGATTTGACCTATTAACAGTCAGATGGGATGCGACTCTTTTGAGGAAA

ATTCGTCATAATGCGAGTCGAGCAAAGCCGGATTGTGTATGTGTGATGTTGGATGTCTCA

TCGCGACCACTCACCATCGCCATCCTTGTCCTGTGCCCGGCGAGTTGGTTAGATACCAAC

GAACTAAAATTGAGTCATTGGCCGTGGTCATAATCAAACTTACAAATAGAGAAAAGGCCT

CTTGGGTACTCGGA

>5048 actin (ACT) gene, partial cds

TATGTGCAAGGCCGGTTTCGCCGGTGACGATGCGCCCCGAGCAGTCTTCCGTAAGTACCTCCATCTCCGCCTTATCAGCCAAGGCTGCATCAAGCGCGATTCGAGAGCACTTTCTGACAGCTCGCAGCTTCCATCGTCGGCCGACCGCGTCACCATGGGTACGATGAACCTCCCGTAATTCCCACGCAATCCGCCATCTAACAACACACAGTATCATGATTGGTATGGGCCAGAAGGACTCGTAA

>5083 actin (ACT) gene, partial cds

TATGTGCAAGGCGGTTTCGCCGGTGATGACGCTCCCCGAGCTGTTTTCCGTGAGTACCCCACTTCTGCTCCTTTCCCACGCAACTGCCCGCCATAGCTATACGTTTTGGGGCTATTGGGCGTTTGCGCGCCACCGCCGCACGCTTCGCTTGACATTTCGCTAACGCCGCCAAACAGCCTCTATTGTCGGTAGACCCCGTCACCATGGGTAAGTTATCATAATCAACACTCACGTGTCGACCGCCGTGATTTTTGGCGGTTGCATCTTGAGAAACATGGATCTGACTGAATCTCCTTCAGTATCATGATTGGTATGGGCCAGAAGGACTCGTAA

>5111 actin (ACT) gene, partial cds

TTACGAGTCCTTCTGGCCCATACCGATCATGATACTGCGTCGCTGTTAGCTCGGGCTCGG

CAAGAGCTGCCGGGGGGAATCGTCATACCCATGGTGACGGGGACGACCGACAATGGAGGC

TGCGTGTGGTCAGATCGAAGTTCTCGGGCGTTCACTGGCGGCAGAGTTGGTGCAATGTAG

TGTTGCTACTGCTGCTGCTGCCAGGTGGCGGGTTGGGGAACTTACGGAAGACTGCTCGGG

GCGCATCATCACCGGCGAAACCGGCCTTGCACATA

>5040 actin (ACT) gene, partial cds

TTACGAGTCCTTCTGGCCCATACCAATCATGATACTGTGTGTTGTTAGATGGCGGATTGC

GTGGGAATTACGGGAGGTTCATCGTACCCATGGTGACGCGGTCGGCCGACGATGGAAGCT

GCGAGCTGTCAGAAAGTGCTCTCGAATCGCGCTTGATGCAGCCTTGGCTGATAAGGCGGA

GATGGAGGTACTTACGGAAGACTGCTCGGGGCGCATCGTCACCGGCGAAACCGGCCTTGC

ACATA

>5259 actin (ACT) gene, partial cds

TTACGAGTCCTTCTGGCCCATACCAATCATGATACTACATTTTGTTATTTAGACCTGACA

TTTGTTTTCTTGGCGTAGTCAAACCCCGCATAGAGAGCAATTGGGGATGACAAGAGGGAG

ACTTACCCATGGTGACGGGGGCGACCAACGATCGAAGCTAGACGTATTATTAGTATGCCA

CGGTTTCATGAAAACGAGTCTGGCGATACTTCTGGATGCAAGGGCACCCGGTGCTATCGC

CAACCTGACTCGGCAGGATCGTGGAAGGCTTGACTTACGGAACACAGCTCGGGGTGCGTC

ATCACCGGCGAAACCGCCTTGCACATAAA
